# Class I and II NADPH-cytochrome P450 reductases exhibit different roles in triterpenoid biosynthesis in *Lotus japonicus*

**DOI:** 10.3389/fpls.2023.1214602

**Published:** 2023-08-09

**Authors:** Pramesti Istiandari, Shuhei Yasumoto, Hikaru Seki, Ery Odette Fukushima, Toshiya Muranaka

**Affiliations:** ^1^ Department of Biotechnology, Graduate School of Engineering, Osaka University, Suita, Japan; ^2^ Industrial Biotechnology Initiative Division, Institute for Open and Transdisciplinary Research Initiatives, Osaka University, Suita, Japan; ^3^ Plant Translational Research Group, Universidad Regional Amazónica IKIAM, Tena, Ecuador

**Keywords:** CRISPR/Cas9, cytochrome P450 monooxygenases (CYP), LORE1, *Lotus japonicus*, NADPH-cytochrome P450 reductases (CPR), triterpenoid biosynthesis

## Abstract

Cytochrome P450 monooxygenases (CYPs) are enzymes that play critical roles in the structural diversification of triterpenoids. To perform site-specific oxidations of the triterpene scaffold, CYPs require electrons transferred by NADPH-cytochrome P450 reductase (CPR), which is classified into two main classes, class I and class II, based on their structural difference. *Lotus japonicus* is a triterpenoids-producing model legume with one CPR class I gene (*LjCPR1*) and a minimum of two CPR class II genes (*LjCPR2-1* and *LjCPR2-2*). CPR classes I and II from different plants have been reported to be involved in different metabolic pathways. By performing gene expression analyses of *L. japonicus* hairy root culture treated with methyl jasmonate (MeJA), this study revealed that *LjCPR1, CYP716A51*, and *LUS* were down-regulated which resulted in no change in betulinic acid and lupeol content. In contrast, *LjCPR2s, bAS, CYP93E1*, and *CYP72A61* were significantly upregulated by MeJA treatment, followed by a significant increase of the precursors for soyasaponins, i.e. β-amyrin, 24-OH β-amyrin, and sophoradiol content. Triterpenoids profile analysis of *LORE1* insertion and hairy root mutants showed that the loss of the *Ljcpr2-1* gene significantly reduced soyasaponins precursors but not in *Ljcpr1* mutants. However, *Ljcpr1* and *Ljcpr2-1* mutants showed a significant reduction in lupeol and oleanolic, ursolic, and betulinic acid contents. Furthermore, *LjCPR1*, but not *LjCPR2*, was crucial for seed development, supporting the previous notion that CPR class I might support plant basal metabolism. This study suggests that CPR classes I and II play different roles in *L. japonicus* triterpenoid biosynthesis.

## 1 Introduction

As one of the model legumes, *Lotus japonicus* is known to accumulate diverse phytochemicals, especially triterpenoid saponins (Suzuki et al., 2019). Triterpenoid saponins are beneficial as anti-cancer and anti-inflammatory agents for humans and are also known to play an essential role as plant defensive compounds against pathogenic bacteria and herbivores. Oxidosqualene cyclases (OSCs) catalyze the first step in triterpenoid biosynthesis by performing the cyclization of 2,3-oxidosqualene to various triterpene scaffolds. Cytochrome P450 monooxygenases (CYPs) are the enzyme most responsible for the structural diversity of triterpenoids due to their capability to perform site-specific oxidations (e.g., the introduction of hydroxyl, ketone, aldehyde, carboxyl, or epoxy groups) on various triterpene skeletons to produce triterpenoid sapogenins (aglycones). The triterpenoid sapogenins are modified with different sugar moieties by glycosyltransferases to produce triterpenoid glycosides, referred to as triterpenoid saponins.

The availability of an *L. japonicus* genome database, mutant library, and the establishment of its hairy root transformation make this plant an excellent platform for studying triterpenoid biosynthesis and its regulatory mechanisms (Urbański et al., 2013). Many triterpenoid biosynthetic genes in *L. japonicus* have been characterized. At least five *L. japonicus* OSCs have been identified, among which are *LjbAS*, *LjAaS*, and *LjLUS*. Also, the functions of three *LjCYP*s involved in triterpenoid biosynthesis, that is, *LjCYP716A51*, *LjCYP72A61*, and *LjCYP93E1*, have been described before (Suzuki et al., 2019). Compared to other tissues, the roots accumulate the highest amount of total triterpenoids in *L. japonicus* plants (Suzuki et al., 2019). Thus, the roots of *L. japonicus* are the best choice for studying triterpenoid biosynthesis.

To perform site-specific oxidation of the triterpene scaffold, CYPs require electrons transferred by its redox partner, NADPH-cytochrome P450 reductase (CPR). Plants have multiple CPR genes, depending on the species, unlike mammals and fungi, which have one CPR gene (Mizutani and Ohta, 1998; Jensen and Møller, 2010). Plant CPRs are branched into two classes, CPR classes I and II (Qu et al., 2015). CPR class I generally has a shorter N-terminal membrane sequence than CPR class II (Parage et al., 2016). CPR class I is reported to be constitutively expressed and plays a role in primary or basal constitutive metabolism, while CPR class II is inducible by environmental stimuli and is involved in defense mechanisms through plant secondary metabolism (Parage et al., 2016). Different tissue expression profiles of CPR classes I and II were reported in *Withania somnifera* (Rana et al., 2013), *Panax ginseng* (Zou et al., 2021), *Camellia sinensis* (Huang et al., 2021), and *Catharanthus roseus* (Parage et al., 2016). The differences in the protein sequences and expression levels of CPR classes I and II suggest that they have different roles in plants.

CPR and CYP are both membrane-bound proteins that have been reported to be present in microsomes in a ratio of 1:15 (Shephard et al., 1983). The significantly low ratio of CPR : CYP implied a competition between a vast number of different CYPs over a small number of CPR, in which systematic regulation is required to perform their functions properly. Knocking down *CPR2* gene in *C. roseus* significantly reduced the total monoterpene indole alkaloid content, while knocking down *CPR1* did not show any change (Parage et al., 2016). ATR2 mutation decreased the electron transfer to three CYPs involved in lignin-related phenolic metabolites, C4H, C3H1, and F5H1, but had small effects on other CYPs involved in glucosinolate and flavonol glycoside biosynthesis of *Arabidopsis thaliana* (Sundin et al., 2014).

Transcript and metabolite profiling of stress/elicitor-treated plants or cell cultures can be used to determine gene function in secondary metabolism (Misra et al., 2014). CPR class II genes from *W. somnifera*, *P. ginseng*, *C. sinensis*, and *C. roseus* were highly induced by methyl jasmonate (MeJA) treatment, whereas their CPR class I genes showed less or no induction (Rana et al., 2013; Parage et al., 2016; Huang et al., 2021; Zou et al., 2021). Interestingly, this different effect of phytohormone treatment was also observed in *OSC*s and *CYP*s. RT-PCR analysis of MeJA-treated *Ocimum basilicum* showed that *ObbAS1* and *ObCYP2* were significantly and continually induced until 12 h of treatment, while the phytohormone effect on *ObbAS2*, *ObCYP1*, and *ObCYP3* was not that apparent (Misra et al., 2014). These results implied that there could also be a differential regulation of plant CPR class in triterpenoid biosynthesis of *L. japonicus* upon MeJA elicitation.

Mutants are a powerful tool for investigating gene functions, including the impact of different CPR classes on *L. japonicus*. Given its status as an extensively studied model legume, several methods have been employed to generate mutants in *L. japonicus*. The Lotus Retrotransposon 1 (*LORE1*) mutant library has been used to study the genome-wide mutagenesis of *L. japonicus*. *LORE1* is a long-terminal repeat of transposable elements induced during tissue culture regeneration in the germ line of *L. japonicus* (Urbański et al., 2013). By tagging *LORE1* elements, a useful mutant library was created. Targeted mutagenesis using the clustered regularly interspaced short palindromic repeats (CRISPR)/CRISPR-associated protein 9 (CRISPR/Cas9) in *L. japonicus* hairy roots has facilitated the investigation of triterpenoid biosynthetic gene function (Suzuki et al., 2019).

Therefore, this study aims to elucidate the role of different classes of LjCPRs in triterpenoid biosynthesis *in planta*. We first analyzed the effect of MeJA treatment on triterpenoid biosynthetic genes and metabolite profiles of *L. japonicus* hairy roots. This study showed that *L. japonicus* NADPH-cytochrome P450 reductase class I (*LjCPR1*) and II (*LjCPR2*) have different regulations upon MeJA addition and revealed different sets of triterpenoid biosynthetic genes co-regulated with either *LjCPR* class I or II. To confirm the involvement of LjCPR classes on these triterpenoids *in planta*, we analyzed the triterpenoid profile of *Ljcpr1* and *Ljcpr2-1 LORE1* insertion mutant plants. We observed different effects on triterpenoid profiles in *Ljcpr1* and *Ljcpr2-1* mutants. To further confirm this result, we generated Δ*ljcpr1* knocked-out hairy root mutants by CRISPR/Cas9, which showed a similar triterpenoid profile with *Ljcpr1 LORE1* insertion mutants. We also observed physiological changes of the *Ljcpr LORE1* insertion mutants, which revealed another possible role of LjCPR classes I and II in other metabolic pathways. This study demonstrated for the first time that CPR classes I and II have different roles in triterpenoid biosynthesis *in planta*.

## 2 Materials and methods

### 2.1 Phylogenetic and gene co-expression analysis of LjCPRs

*LjCPR* gene sequences were obtained from Miyakojima MG-20 (Li et al., 2020) and the Gifu v1.2 genome database (https://lotus.au.dk/expat/) by using BLAST with *A. thaliana* CPR1 and CPR2 as the query. Phylogenetic analysis on LjCPRs was conducted using 29 other CPR sequences from different plant species, which were obtained from the web-based resource for *Arabidopsis* P450, cytochrome *b*, NADPH-cytochrome P450 reductases, and family 1 glycosyltransferases (www.P450.kvl.dk), NCBI (https://www.ncbi.nlm.nih.gov/), and various plant genome databases. The classification of CPR classes I and II was based on *A. thaliana* CPR1 and CPR2, as previously reported (Mizutani and Ohta, 1998). Accession numbers of CPR genes and genome databases used in this study are listed in **Supplementary Table S1**. Multiple amino acid sequences were aligned using ClustalW and were used for tree construction using the neighbor-joining method with MEGA7. Gene co-expression analysis of *L. japonicus* was performed using an online transcriptomic database from the gene expression atlas web server https://lotus.au.dk/expat/ using the Gifu v1.2 genome database version. Based on the sequence identity between CPR genes of Miyakojima MG-20 and Gifu ecotype, gene IDs LotjaGi1g1v0345200.1, LotjaGi4g1v0301400.3, and LotjaGi4g1v0301300.1 from Gifu genome database were used for *LjCPR1*, *LjCPR2-1*, and *LjCPR2-2* genes in this study (**Supplementary Table S2**).

### 2.2 Plant materials and germination treatment

*L. japonicus* Gifu B-129 wild-type (WT) and *LORE1* insertion lines (Fukai et al., 2012; Urbański et al., 2012) were provided by Miyazaki University, Japan, and Aarhus University, Denmark, through the National BioResource Project (NBRP). Seeds of *L. japonicus* were surface-sterilized using 2% (v/v) sodium hypochlorite and 0.02% (v/v) Tween 20 for 15 min in a seesaw shaker, rinsed three times with ultrapure water obtained from a Milli-Q Synthesis system (Millipore, Burlington, MA, USA), and placed onto a 0.8% agar plate. The seeds were allowed to germinate at 23°C for 4 days in the dark and 2 days under a 16-h light:8-h dark photoperiod.

### 2.3 Chemicals

β-Amyrin, α-amyrin, lupeol, erythrodiol, uvaol, oleanolic acid, ursolic acid, and asiatic acid were purchased from Extrasynthese (Genay, France). Betulin and MeJA were purchased from Sigma-Aldrich (St. Louis, MO, USA). Betulinic acid was purchased from Tokyo Chemical Industry (Tokyo, Japan). Soyasapogenol B and soyasapogenol A were purchased from Tokiwa Phytochemical (Chiba, Japan). Sophoradiol, 24-hydroxy-β-amyrin, and soyasapogenol E were kindly provided by Dr. Kiyoshi Ohyama (Tokyo Institute of Technology, Japan).

### 2.4 Hairy root induction

Induction of hairy roots was performed as reported previously (Suzuki et al., 2019), with slight modifications. *Agrobacterium rhizogenes* ATCC15834 strains were cultured on YEB plates for 2 days and suspended in sterilized water. The roots of 7-day-old WT seedlings were cut off, and *A. rhizogenes* were infected into the cross-sections of hypocotyls. After co-cultivation for 4 days, the infected seedlings were cultured on cefotaxime-containing hairy root elongation (HRE) solid medium for 2 weeks under a 16-h light:8-h dark photoperiod. After dissection, hairy roots were cultured under dark conditions. The root tip of a randomly chosen healthy WT hairy root clone was subcultured in 5 mL of cefotaxime-containing HRE liquid medium for 2 weeks and then transferred to 5 mL of HRE liquid medium without antibiotics with shaking at 90 rpm for another 2 weeks. Isolated hairy roots were cultured for 2 months at room temperature with subculturing every 3–4 weeks. Finally, hairy roots were cultured in 100 mL of HRE liquid medium at 25°C with shaking at 90 rpm for 4 weeks.

### 2.5 Methyl jasmonate preparation and addition

MeJA elicitation was conducted to test its effect on triterpenoid biosynthesis in *L. japonicus* hairy roots. MeJA preparation was performed as reported previously (Akhgari et al., 2019). A 20-mM MeJA stock solution was made by dissolving it in 40% (v/v) ethanol and then was filter sterilized (0.22 µm). The 4-week culture of WT hairy root was cut into similar portions (roughly 200 mg fresh weight each), cultured into 100 mL of HRE liquid medium without antibiotics, and incubated at 25°C with shaking at 90 rpm for another 4 weeks. The 4-week-old hairy root cultures were supplemented with final concentrations of 100 µM of MeJA. For control cultures, equal volumes (500 µL) of 40% ethanol were added to 100 mL of culture medium. The hairy roots were incubated under the same conditions as mentioned above and collected after 0, 3, 6, and 12 h for gene expression analysis and 0, 12, 24, and 24 h for metabolite analysis. The samples were flash-frozen in liquid nitrogen and stored at −80°C until use.

### 2.6 Quantitative real-time PCR

Total RNA was extracted from 100 mg of 4-week-old frozen *L. japonicus* MeJA-treated and control hairy roots using RNeasy Plant Mini Kit (Qiagen, Germantown, MD, USA). The RNA obtained was purified using the After Tri-Reagent RNA Clean-Up Kit (Favorgen Biotech Corp., Ping Tung, Taiwan) after digesting contaminated genomic DNA with recombinant DNase I (RNase-free) (TaKaRa Bio, Shiga, Japan). First-strand cDNA was synthesized from purified total RNA by PrimeScript RT Master Mix (Perfect Real Time) (TaKaRa Bio). qPCR analysis was performed using The LightCycler^®^ 96 (Roche, Basel, Switzerland) and FastStart Essential DNA Green Master (Roche). The primers used for qPCR analysis are listed in **Supplementary Table S3**. The expression of ubiquitin (UBQ) gene was analyzed as a reference gene.

### 2.7 *Ljcpr1* and *Ljcpr2* loss-of-function mutant lines

The loss-of-function mutant lines used in this study were the *LORE1* mutant collection with Gifu B-129 genetic background annotated into the Miyakojima MG-20 v3.0 genome assembly. *LjCPR1* and *LjCPR2-1* were mapped into gene IDs Lj1g3v1548790.1 and Lj4g3v2107220.1 in the Miyakojima MG-20 v3.0 genome version, respectively. Several *LORE1* lines mapped to each gene ID were selected from *LORE1* mutant library (lotus.au.dk) and screened to obtain homozygous mutants. Two independent lines of *Ljcpr1* (30003941 and 30059903) and *Ljcpr2-1* (30037476 and 30065390) homozygous insertion mutants were obtained. Each mutant line contains other exonic or intronic *LORE1* insertions other than *LjCPR* genes (**Supplementary Table S4**). These chosen mutant lines were cultivated in soil, and their progenies were cultivated in a hydroponic system.

For soil-cultured plants, the 7-day-old WT and mutant seedlings were moved to pots with a mixture of soil and vermiculite and cultivated for 3 months. The produced seeds were then collected for hydroponic cultivation. The seed pods from three independent lines for each *Ljcpr1* and *Ljcpr2-1* homozygous insertion mutant were counted. Then, the pod length of 26 randomly selected pods from each *Ljcpr1* and *Ljcpr2-1* homozygous insertion mutant line was measured using a digital vernier caliper. The 7-day-old WT and mutant seedlings of the soil-cultured plant progenies were first cultured in 5-mL tubes containing basal nutrient solution. After 2 weeks, the hydroponic plants were scaled up into 50-mL tubes containing basal nutrient solution and cultivated until the flowering stage. The hydroponic medium was renewed weekly.

Genomic DNA was extracted and purified from the leaves using FavorPrep™ Plant Genomic DNA Extraction Mini Kit (Favorgen Biotech Corp.) to screen for homozygous *Ljcpr1* and *Ljcpr2-1* mutants using PCR with CPR-specific primers and a *LORE1*-specific primer (**Supplementary Table S5**; **Supplementary Figure S1**). PCR was performed using KOD FX Neo following the manufacturer’s instructions (Toyobo, Osaka, Japan) using the same DNA concentration for all samples. Triterpenoids were extracted from the soil- and hydroponic-cultured roots of homozygous and heterozygous mutants and were analyzed as described below.

### 2.8 Generation of *Ljcpr1* knockout mutant hairy root lines

The multiplex guide RNA (gRNA)-expressing CRISPR-Cas9 vector, pMgP237-2A-GFP (Hashimoto et al., 2018), was used for genome editing of *L. japonicus* hairy root. The target sequences of the gRNAs (**Supplementary Figure S2**) were selected from *LjCPR1* gene using the web-based tool CRISPRdirect (https://crispr.dbcls.jp/) (Naito et al., 2015). Two gRNA target sequences were simultaneously transferred into the pMgP237-2A-GFP vector as described previously (Nakayasu et al., 2018), generating the T1/T2-pMgP237 vector. Three sets of different gRNA designs (Nos. 2, 4, and 5) targeting *LjCPR1* gene were constructed using the primers listed in **Supplementary Table S6**. *A. rhizogenes* ATCC15834 was transformed with the pMgP237 empty vector or the T1/T2-pMgP237 vector.

The induction of hairy roots was described above. Crude genomic DNA extraction and PCR were performed using KOD FX Neo following the manufacturer’s instructions (Toyobo). Mutagenesis was confirmed using PCR with specific primers for each gRNA design (**Supplementary Table S6**; **Supplementary Figure S3**) and analyzed using a heteroduplex mobility assay (HMA) and the MCE-202 MultiNA microchip electrophoresis system (Shimadzu, Kyoto, Japan) following the manufacturer’s instructions. The target sequences amplified from putative mutants were cloned into the pJET1.2/blunt vector (CloneJET PCR Cloning Kit; Thermo Fisher Scientific, Waltham, MA, USA). Insertion and deletion mutations were confirmed by sequencing several randomly selected clones.

Multiple sequences of nucleotides and amino acids were aligned using MEGA11 software (Tamura et al., 2021). The protein structure of *LjCPR1* from wild-type and L1-4.2 mutant lines was modeled using the SWISS-MODEL (Arnold et al., 2006). The LjCPR1 models were constructed using two crystal structure templates, A0A0R4J338.1.A (NADPH-cytochrome P450 reductase of *Glycine max*) and 5GXU.1.A (NADPH-cytochrome P450 reductase 2 of *A. thaliana*), with 87.7% and 72.8% identity, respectively. These two templates were used due to the different properties exhibited by the crystal structures. The crystal structure of A0A0R4J338.1.A presents the complete protein structure of *G. max* CPR, including the transmembrane region. Meanwhile, 5GXU.1.A is a truncated *A. thaliana* CPR crystal structure without the transmembrane region and includes the position of the covalently bonded flavin mononucleotide (FMN) ligand on the CPR protein. The protein models were then visualized using PyMOL (DeLano, 2020) to compare the conformations of the wild-type and mutant proteins.

### 2.9 Triterpenoid extraction from *L. japonicus* plants and hairy roots

Triterpenoid extraction was performed as reported previously (Suzuki et al., 2019), with slight modifications. Plants at the flowering stage and hairy roots were lyophilized and powdered using a multi-bead shocker (Yasui Kikai, Osaka, Japan). Powdered tissues (20.00 ± 0.3 mg) were extracted three times with 1 mL of methanol using a sonication-assisted method. Completely dry extracts were resuspended in 2 mL of MeOH in 4 M of HCl (1:1). The extracts were incubated at 80°C for 1 h to remove the sugar moieties of triterpenoid saponins. The hydrolyzed products were extracted three times with hexane:EtOAc (1:1) and dried completely. The obtained pellet was resuspended in 500 μL of MeOH:chloroform (1:1). A portion of the solution was dried in a gas chromatography–mass spectrometry (GC-MS) vial. Additionally, 100 μL of the solution was evaporated and trimethylsilylated using a mixture of 50 μL of *N*,*N*-dimethylformamide (Kishida Chemical Co., Ltd., Osaka, Japan) and 50 μL of BSTFA : TMCS (99:1) (TCI) at 80°C for 30 min. For semi-quantitative analysis, an asiatic acid authentic standard was applied to the plant tissue powder before extraction.

### 2.10 GC-MS analysis

GC-MS analyses were performed as reported previously (Suzuki et al., 2019) on a 5977A MSD mass spectrometer (Agilent Technologies, Santa Clara, CA, USA) connected to a 7890B gas chromatograph (Agilent Technologies) with an HP-5MS UI (30 m × 0.25 mm, 0.25-μm film thickness; Agilent Technologies) capillary column for qualitative analysis. The injection temperature was set at 250°C. The column temperature program was as follows: 80°C for 1 min, increase to 300°C at a rate of 20°C/min, and hold for 28 min. The carrier gas was helium at a flow rate of 1.0 mL/min. The ion source temperature was 230°C, and the quadrupole temperature was 150°C. The derivatized sample (1 µL) was injected in splitless injection mode. Peaks were identified by comparing their Rt and mass spectra with those of authentic standards (**Supplementary Figure S4**). Samples were analyzed in selected ion monitoring (SIM) mode for relative quantification by extracting the mass chromatogram in respective extracted ion chromatogram (EIC) for each metabolite as listed in **Supplementary Table S7**.

### 2.11 Statistical analysis

The significance of differences was determined using a one-way single-factor analysis of variance (ANOVA). The significance of the means was separated using Tukey’s test. p-Values less than 0.05 (p < 0.05) were considered significant in this study. All the statistical analyses were performed with JASP (JASP Team 2020).

## 3 Result

### 3.1 Phylogenetic and gene co-expression analyses of *L. japonicus* CPRs

The two ecotypes of *L. japonicus* most commonly used for research are Miyakojima MG-20 and Gifu B-129 (Hashiguchi et al., 2011). The most recent Miyakojima MG-20 transcripts submitted by Li et al. (2020) showed that *L. japonicus* has one copy of CPR class I gene and two copies of CPR class II genes (**Supplementary Figure S5-A**). This latest genome version of Miyakojima MG-20 was obtained using the Illumina HiSeq 2500 platform and PacBio sequencing system (Li et al., 2020). Interestingly, based on a high-quality *L. japonicus* Gifu v1.2 genome database constructed using 100× PacBio read coverage and RNA-seq analysis (Kamal et al., 2020), seven CPR genes were annotated. One CPR gene belonged to CPR class I, while six CPR genes belonged to CPR class II (**Supplementary Figure S5-B**). In both ecotype genomes, CPR class I gene was located on chromosome 1, while several copies of CPR class II genes were located close to each other on the same locus of chromosome 4 (**Supplementary Figure S5**).

Based on phylogenetic tree analysis, LjCPR class I genes from Miyakojima MG-20 and Gifu B-129 were in the same clade, while the LjCPR class II genes from Miyakojima MG-20 and Gifu B-129 branched into two different clades, namely, LjCPR2-1 and LjCPR2-2 (**Supplementary Figure S6**). Two copies of the CPR class II in Miyakojima MG-20 genome belonged to two different LjCPR2 clades. However, from the six LjCPR class II genes found in the Gifu genome, three genes belonged to the LjCPR2-1 clade, and three genes belonged to the LjCPR2-2 clade. A multiple sequence alignment analysis and amino acid and nucleotide sequence identity matrix were constructed to evaluate homology among *LjCPR* genes (**Supplementary Figure S7**; **Supplementary Tables S2A, B**). LjCPRs showed that LjCPR class I from both ecotypes had 100% nucleotide similarity (**Supplementary Tables S2A, B**). All *LjCPR2-2* genes from Gifu showed 100% nucleotide similarity, but they are not identical to Miyakojima MG-20 *LjCPR2-2*, with 99.1% amino acid similarity (**Supplementary Tables S2A, B**). In contrast, one isoform of LjCPR2-1 from Gifu (LotjaGi4g1v0301400.3) showed a sequence identical to that of LjCPR2-1 from Miyakojima (**Supplementary Table S2B**).

**Figure 1:**
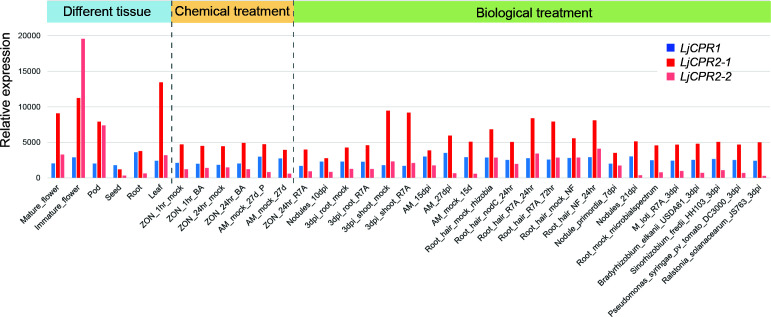
Gene co-expression analysis of *L. japonicus* using transcriptomic data from 35 different samples from different tissues, chemical treatments, or biological treatments. The transcriptomic data were obtained from the Gifu genome assembly v1.2 (https://lotus.au.dk/ of).

Transcriptomic data were retrieved from the database to analyze the gene expression level of different CPR classes in *L. japonicus*. Unfortunately, the transcriptomic database was unavailable for the *L. japonicus* Miyakojima MG-20 genome (Li et al., 2020). Therefore, in this study, only the expression level of *LjCPR* genes from the Gifu v1.2 transcriptomic database was analyzed. *LjCPR1* was mapped to probe ID LotjaGi1g1v0345200. *LjCPR2-1* was mapped to probe ID LotjaGi4g1v0301400. The identical sequence of Gifu *LjCPR2-2s* was mapped to the LotjaGi4g1v0301300 probe, labeled as *LjCPR2-2* in this study. CPR class I and II genes of *L. japonicus* showed distinct expression patterns (**Figure 1**). As reported in our study before, based on Miyakojima MG-20 v3.0 transcriptomic database, CPR class I was found to be constitutively expressed with lower and more stable expression levels. In contrast, CPR class II was generally found to have higher expression levels than CPR class I, which varied depending on tissues and treatments (Istiandari et al., 2021). Similarly, in the *L. japonicus* Gifu v1.2 transcriptomic database, both *LjCPR2-1* and *LjCPR2-2* showed higher inducible expression than *LjCPR1* (**Figure 1**). However, *LjCPR2-2* expression was even lower than *LjCPR1* expression in some of the samples but seemed to be tissue-specific. Interestingly, *LjCPR2-2* was predominantly expressed in immature flowers and showed the highest expression level among the three CPR genes. Nevertheless, *LjCPR2-1* expressions were generally higher in almost all samples than those of *LjCPR2-2* and *LjCPR1*, implying *LjCPR2-1* is the dominant LjCPR class II in *L. japonicus*.

### 3.2 Effect of MeJA treatment on triterpenoid biosynthesis in *L. japonicus* hairy roots

**Figure 2:**
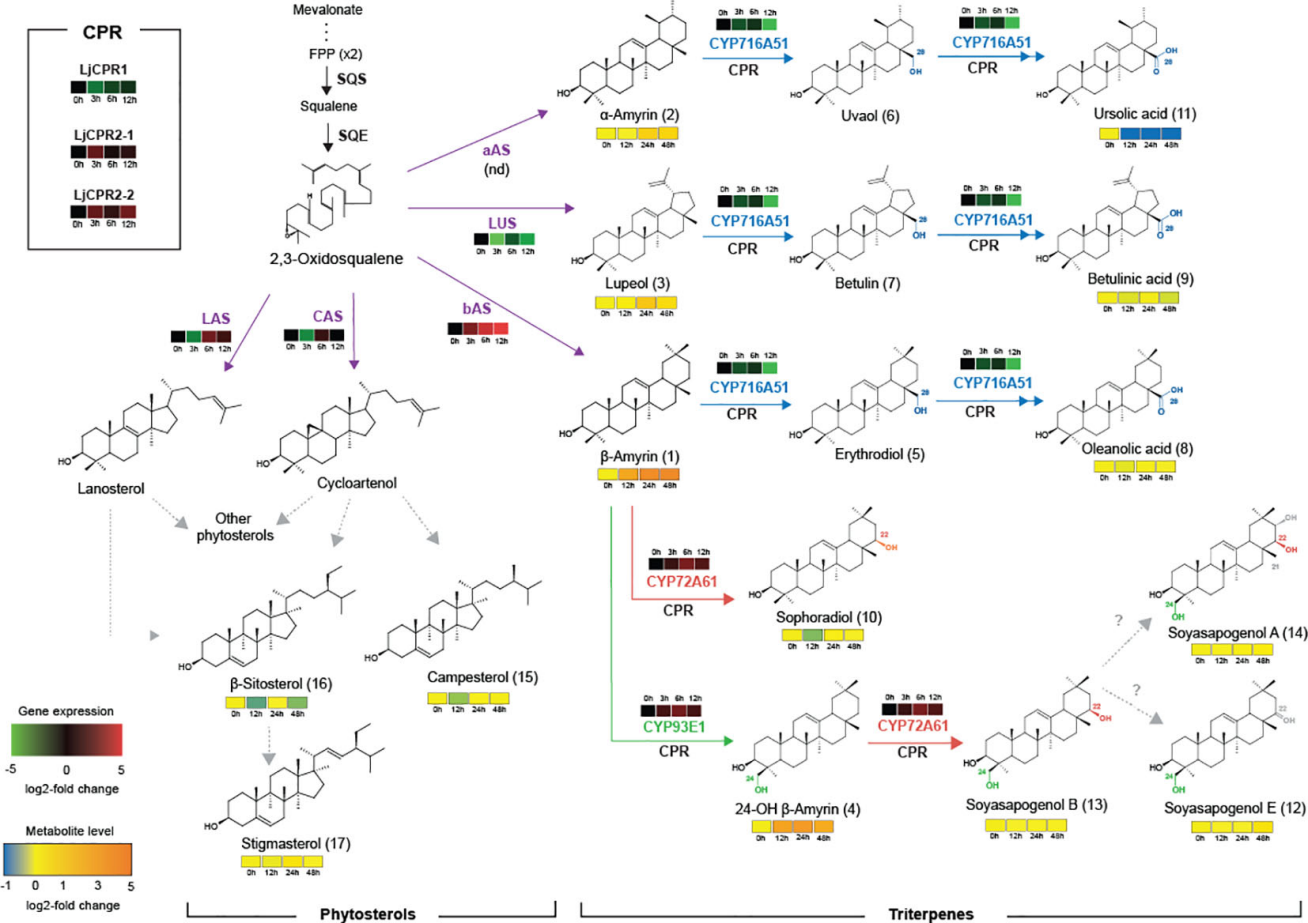
Effect of the addition of 100 μM MeJA sampled at different time points on gene expression and metabolite level on triterpenoids and phytosterol biosynthesis in *L. japonicus* hairy root. Single and double arrows indicate one and two oxidation steps, respectively. Dashed arrows indicate multiple steps. The number in parentheses next to the metabolite name refers to the chromatogram peak in **Supplementary Figure S8**. CYP, cytochrome P450; FPP, farnesyl pyrophosphate; SQS, squalene synthase; SQE, squalene epoxidase; bAS, β-amyrin synthase; aAS, α-amyrin synthase; LUS, lupeol synthase; LAS, lanosterol synthase; CAS, cycloartenol synthase. nd, not detected.

Triterpenoid biosynthetic pathway in *L. japonicus* is shown in **Figure 2**. The major pentacyclic triterpene backbones, β-amyrin, α-amyrin, and lupeol, are converted from 2,3-oxidosqualene by bAS, aAS, and LUS, respectively (**Figure 2**). The carboxylation of β-amyrin, α-amyrin, and lupeol at the C-28 position by CYP716A51 leads to oleanolic acid, ursolic acid, and betulinic acid production (Suzuki et al., 2019). CYP93E1 catalyzes hydroxylation at the C-24 position of β-amyrin to produce 24-hydroxy β-amyrin (Shibuya et al., 2006; Seki et al., 2008). CYP72A61 converts β-amyrin into sophoradiol by adding a hydroxyl group at the C-22 position (Fukushima et al., 2011). 24-Hydroxy β-amyrin is further oxidized by CYP72A61 that catalyzes hydroxylation at the C-22 position, producing the major soyasaponin aglycone soyasapogenol B (Fukushima et al., 2011).

**Figure 3:**
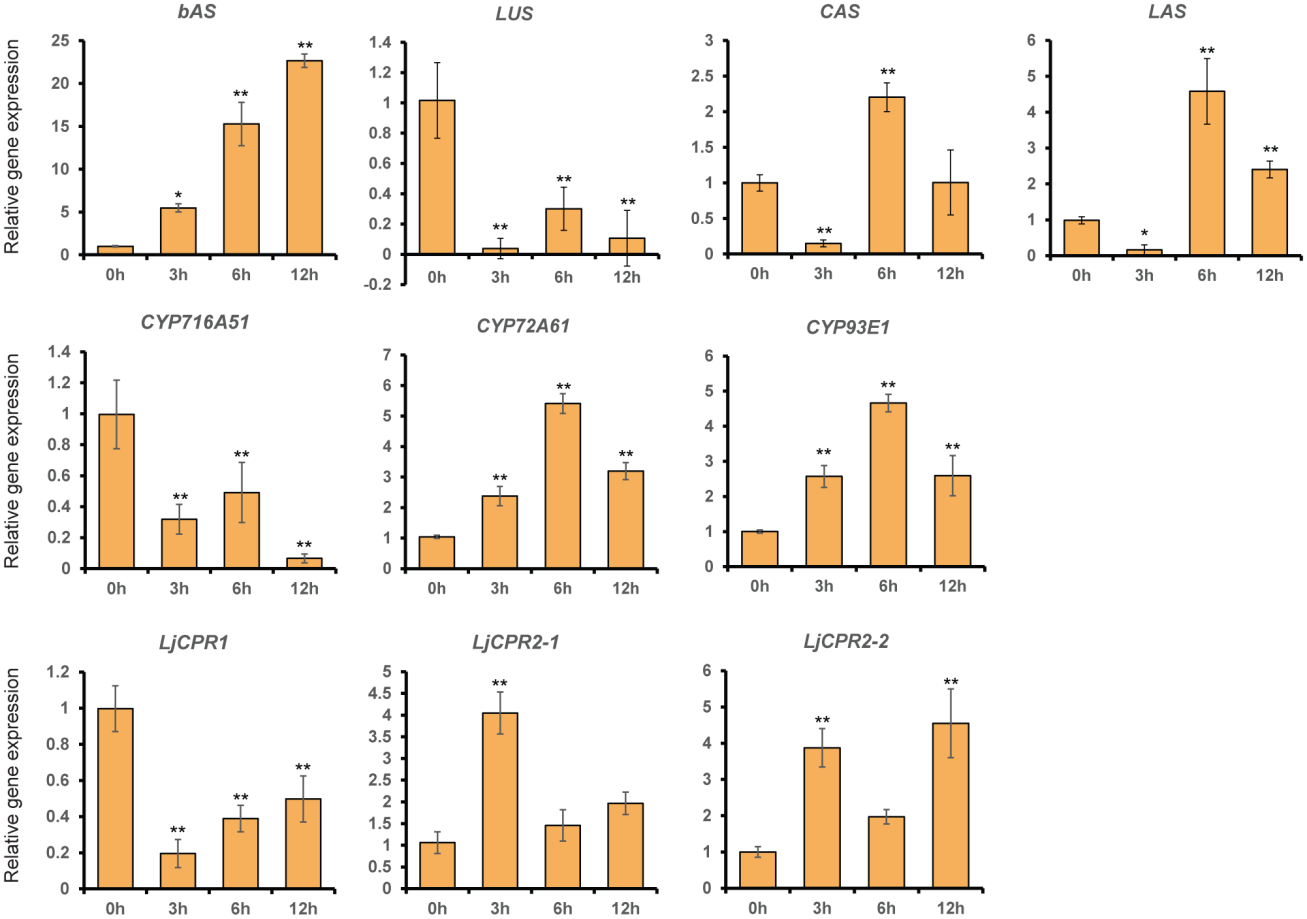
The relative expression of triterpenoid biosynthetic genes in *Lotus japonicus* hairy roots at different time periods after methyl jasmonate (MeJA) treatment. Transcript levels of *LjCPR1*, *LjCPR2-1*, *LjCPR2-2*, *bAS*, *LUS*, *CAS*, *LAS*, *CYP716A51*, *CYP72A61*, and *CYP93E1* were analyzed by qRT-PCR in *L. japonicus* hairy roots treated with 100 μM of MeJA for 0, 3, 6, and 12 h after the treatment. Relative expression levels were normalized to those of ubiquitin and are presented as fold induction relative to the control. Data represent the mean of three independent replicates ± SD. Single-factor ANOVA with Tukey’s *post-hoc* test was used for statistical comparison with the control sample (0 h). Values were considered statistically significant at * p < 0.05 and ** p < 0.01. SD, standard deviation.

MeJA (100 μM) was added to 1-month-old *L. japonicus* hairy roots that were sampled at different time points to elucidate the effect of phytohormone elicitation on the *L. japonicus* triterpenoid biosynthetic pathway (**Figure 2**). *L. japonicus* accession Gifu B-129 was used in this study. The qRT-PCR result of extracted RNA from these treated hairy roots showed differences in the regulation of some triterpenoid biosynthetic genes (**Figure 3**). While *LjCPR1* gene was quickly downregulated by MeJA addition, *LjCPR2-1* and *LjCPR2-2* expressions were significantly upregulated up to four times when compared to the mock sample 3 h after MeJA addition. Interestingly, similar to the *LjCPR1* expression pattern, *CYP716A51* and *LUS* genes also showed downregulation by MeJA addition even 12 h after the treatment. However, very high and quick upregulation was observed in *bAS*, *CYP93E1*, and *CYP72A61* expression levels. The expression of *bAS* was upregulated more than 20 times when compared to the control 12 h after the treatment, while *CYP72A61* and *CYP93E1* upregulation was the highest 6 h after the treatment at approximately five times higher than those of the control. Another triterpene *OSC* gene, *aAS*, was not detected in all samples. To observe the effect of MeJA on primary metabolites such as phytosterols, *CAS* and *LAS* expressions were also analyzed. *CAS* and *LAS* are cycloartenol and lanosterol synthase, respectively, which represent the branch-off entry of phytosterol biosynthesis after 2,3-oxidosqualene cyclization (**Figure 2**). Both *CAS* and *LAS* expressions were instantly downregulated 3 h after MeJA addition, but then the expressions increased after 6 h and returned to levels similar to those of the control 12 h after the treatment (**Figure 3**).

**Figure 4:**
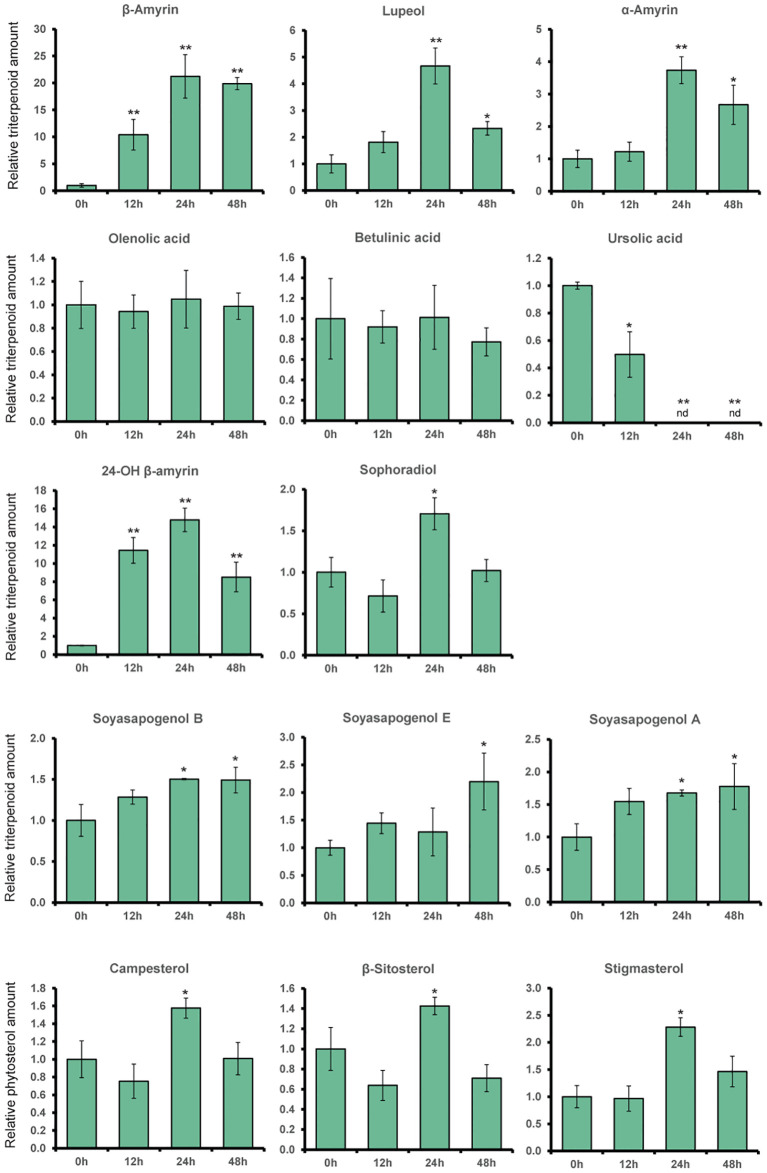
The relative amount of triterpenoids and phytosterol content of *L. japonicus* hairy roots treated with methyl jasmonate (MeJA) on different time periods. Production levels of α-amyrin, β-amyrin, lupeol, ursolic acid, oleanolic acid, betulinic acid, sophoradiol, 24-OH β-amyrin, soyasapogenols, and phytosterols were analyzed by GC-MS in *L. japonicus* hairy roots treated with 100 μM MeJA for 0, 12, 24, and 48 h after the treatment. Relative triterpenoids and phytosterol amounts were normalized to that of asiatic acid as the internal standard and are presented as fold induction relative to the control. Data represent the mean of three biological replicates ± SD. Single-factor ANOVA with Tukey's post-hoc test was used for statistical comparison with the control sample (0h). Values were considered statistically significant at *P < 0.05 and **P < 0.01. SD, standard deviation. nd, not detected.

Based on GC-MS analysis, the change in expression levels of triterpenoid biosynthetic genes due to MeJA treatment affected the triterpenoid production in *L. japonicus* hairy roots (**Figures 2**, **4**). The triterpenoids analyzed in this study were annotated with numbers corresponding to the chromatogram peaks of MeJA-treated hairy root extracts shown in **Supplementary Figure S8**. Consistent with the significant upregulation of *bAS* and *CYP93E1* expressions (**Figure 3**), MeJA addition resulted in a significant increase in β-amyrin and 24-OH β-amyrin levels (**Figure 4**). The β-amyrin level was increased 10 times 12 h after the treatment and continued to increase until it reached 20 times higher than that of the control even 48 h after the treatment. Similar to β-amyrin level, the 24-OH β-amyrin level also increased rapidly after 12 h to 10 times higher than that of the control and reached a maximum of 24 h after the treatment at 15 times higher than that of the control. Sophoradiol production level also increased 24 h after the treatment. In addition, soyasapogenols showed gradually increasing production levels even 48 h after the treatment. However, no change in oleanolic acid and betulinic acid levels, and even a significant reduction of the ursolic acid level, was observed upon MeJA treatment. However, the lupeol level showed a significant increase after 24 h up to four times that of the control, similar to α-amyrin production. All triterpenoids’ peaks were annotated, and the chromatogram area was measured by comparing the retention time and mass spectrum with their authentic standards. The analyzed triterpenoids were the major triterpenoid constituents in *L. japonicus* (Suzuki et al., 2019), which served as the representative for triterpenoids in this study. The levels of other minor triterpenoids were too low to be detected by GC-MS. Campesterol, β-sitosterol, and stigmasterol, as the three major sterols in plants, were analyzed to observe the effect of MeJA on the phytosterol pathway. Due to the lack of standard compounds, the phytosterol peak was annotated based on similarity with a mass spectrum from the NIST library (**Supplementary Figure S9**). All phytosterols showed only a small increase after 24 h and returned to levels similar to those of the control after 48 h (**Figure 4**).

### 3.3 *Ljcpr1* and *Ljcpr2-1* loss-of-function mutant plants

**Figure 5:**
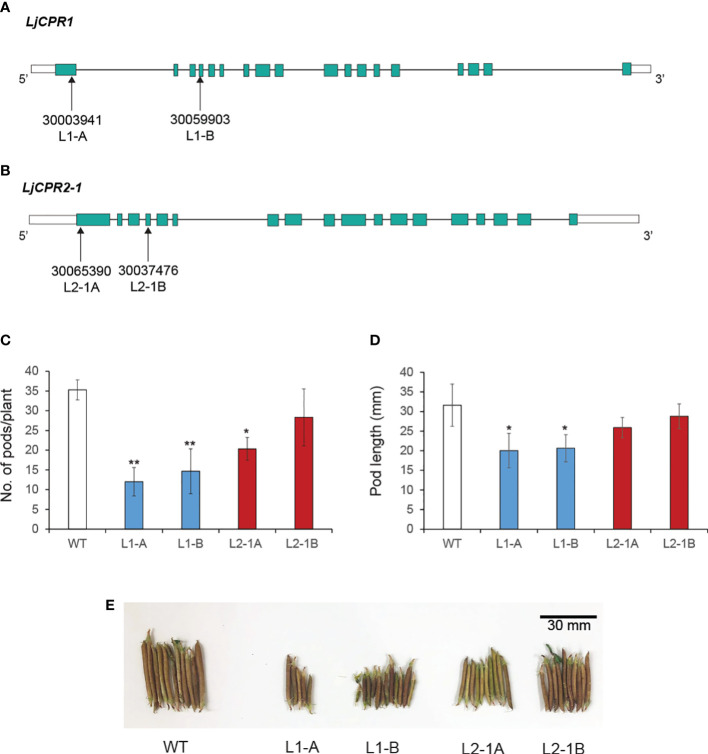
The *LORE1* insertion mutant lines selected for this study. Two homozygous *LORE1* insertion mutant lines were chosen as **(A)** *Ljcpr1* and **(B)** *Ljcpr2-1* loss-of-function mutants. The **(C)** pod numbers and **(D)** length of each mutant were quantified. The photo of representative mutant pods is shown in panel **(E)** Data represent the mean of three biological replicates in pod count **(C)** and N = 26 randomly selected pods from each mutant plant for the pod length measurement **(D)**, and both are presented as ± SD. Single-factor ANOVA with Tukey’s *post-hoc* test was used for statistical comparisons. Values were considered statistically significant at * p < 0.05 and ** p < 0.01. SD, standard deviation.

The triterpenoid profiles of *LORE1* insertion mutant lines of *L. japonicus* accession Gifu B-129 (Fukai et al., 2012; Urbański et al., 2012) were analyzed to investigate the effect of loss-of-function of either *LjCPR1* or *LjCPR2* genes. Based on a previous study, *LUS* and *CYP716A*s genes that are responsible for lupeol and betulinic acid production, respectively, were upregulated in secondary aerenchyma of hydroponic-cultured *L. japonicus* (Suzuki et al., 2022) and soybean plants under flooded conditions (Takahashi et al., 2022). Therefore, to investigate if plant culture condition affects LjCPRs’ involvement with specific CYPs, the *Ljcpr1* and *Ljcpr2-1* loss-of-function mutant lines were both cultured in soil and a hydroponic system. The seeds of two independent homozygous mutant lines, 30003941 (L1-A) and 30059903 (L1-B), which contain a single *LORE1* insertion into the first and fourth exons of *LjCPR1*, were obtained (**Figure 5A**). Other seeds of two independent homozygous mutant lines 30037476 (L2-1A) and 30065390 (L2-1B), which contain a non-single insertion *LORE1* insertion into the first and fourth exons of *LjCPR2-1*, were obtained (**Figure 5B**).

While both *Ljcpr1* mutants are single insertion homozygous mutants (**Supplementary Figure S1**), PCR genotyping results showed that there were heterozygous insertions on other genes other than *LjCPR2-1* in these mutant lines (**Supplementary Figure S1**). Therefore, the effect of other gene mutations on both *Ljcpr2-1* mutant lines should not be ruled out. Other seeds of two independent mutant lines, which contain a *LORE1* insertion on the *LjCPR2-2* exon, were also screened; however, no homozygous mutant was obtained. Therefore, only *Ljcpr1* and *Ljcpr2-1* loss-of-function mutants were analyzed in this study. *Ljcpr2-1* mutant lines might serve as a representative mutant line for LjCPR class II since they were more dominantly expressed than *LjCPR2-2* (**Figure 1**). Some WT allele-specific primer sets (F and R) and insertion allele-specific primer sets (F and P2) were designed for *LjCPR1* and *LjCPR2-1* genes and other genes that may have *LORE1* insertions in their exons (**Supplementary Figure S1**; **Supplementary Table S2**) according to a previous report (Urbański et al., 2012).

No significant difference in the physiology of the *Ljcpr* mutants could be observed in the plant leaves, stems, or roots. However, a notable change was observed in the seed pods (**Figures 5C–E**). The collected seed pods from soil-cultured mutants were then measured and counted to observe the effect of *Ljcpr* loss on the seed physiology (**Figures 5C, D**). The number of pods and pod length of two homozygous *Ljcpr1* mutant lines showed a more significant reduction compared to *Ljcpr2-1* mutant seed pods (**Figure 5D**). The physiological change of the seed pod can be observed in **Figure 5E**.

**Figure 6:**
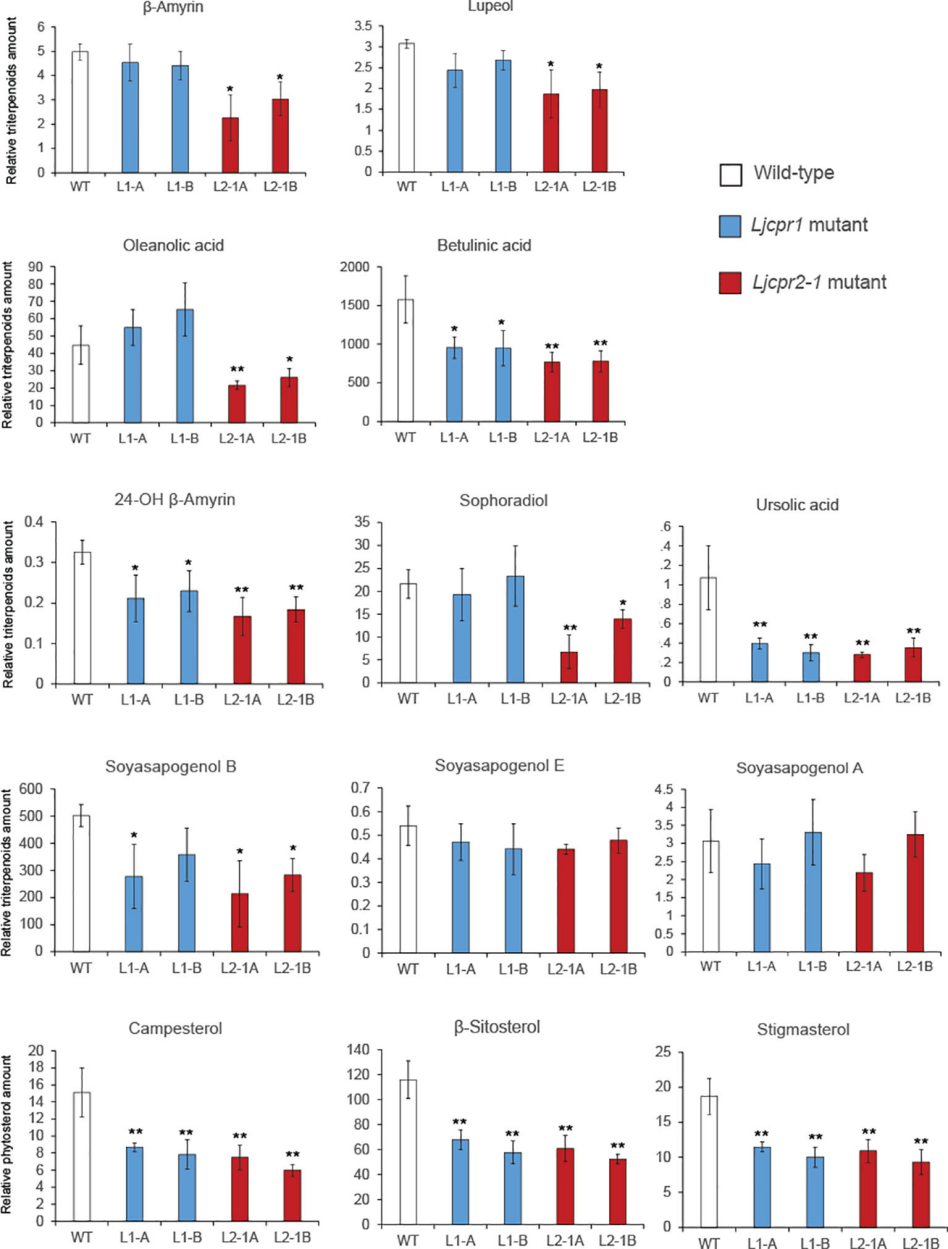
The relative amount of triterpenoids and phytosterols of soil-cultured *Ljcpr LORE1* insertion mutant roots analyzed by GC-MS. Relative triterpenoid and phytosterol amounts were normalized to those of asiatic acid as internal standard and are presented as fold induction relative to the wild-type control (WT). Data represent the mean of three biological replicates ± SD. Single-factor ANOVA with Tukey’s *post-hoc* test was used for statistical comparison to control (WT). Values were considered statistically significant at * p < 0.05 and ** p < 0.01. SD, standard deviation; GC-MS, gas chromatography–mass spectrometry.

The triterpenoid sapogenin profile of each soil-cultured mutant plant was analyzed using GC-MS, and the result showed different profiles for *Ljcpr1* and *Ljcpr2-1* loss-of-function mutant lines (**Figure 6**), similar to that of the hydroponic-cultured mutants (**Supplementary Figure S10**). A significant difference was shown both in soil-cultured and hydroponic-cultured mutant plants. β-Amyrin, oleanolic acid, lupeol, 24-OH β-amyrin, and sophoradiol levels were significantly decreased in *Ljcpr2-1* mutant lines but showed little or no change in *Ljcpr1* mutants (**Figure 6** and **Supplementary Figure S10**). Interestingly, both *Ljcpr1* and *Ljcpr2-1* mutant lines showed significant decreases in betulinic acid and ursolic acid and no change in soyasapogenol E and A (**Figure 6** and **Supplementary Figure S10**). Both mutants showed lower levels of soyasapogenol B. However, the decrease in soyasapogenol B was more significant in hydroponic-cultured (**Supplementary Figure S10**) than in the soil-cultured mutant roots (**Figure 6**). As the three major phytosterols, the campesterol, β-sitosterol, and stigmasterol levels were analyzed and annotated based on mass spectra from the NIST library to investigate the effect of *Ljcpr* loss-of-function on the primary metabolisms (**Supplementary Figure S9**). However, due to a shift in retention time, campesterol could not be detected in hydroponic-cultured samples. Campesterol, β-sitosterol, and stigmasterol were shown to be significantly reduced in both *Ljcpr1* and *Ljcpr2-1* loss-of-function soil-cultured mutants (**Figure 6**).

### 3,4 Knockout of *Ljcpr1* gene in transgenic hairy roots

To directly confirm the involvement of different LjCPR classes on the triterpenoid biosynthetic pathway, we used the CRISPR-Cas9 system. We previously described a CRISPR-Cas9 vector, pMgP237-2A-GFP, which has been used to generate *L. japonicus* hairy root knockout mutants (Suzuki et al., 2019). Since *LjCPR2-1* and *LjCPR2-2* genes have very similar sequence identities, it was very difficult to obtain single or complete null-mutant of double-knockout *Ljcpr2*s due to a lower probability of removing all the intact sequences. Thus, in this study, only *Ljcpr1* knockout mutants were successfully obtained and analyzed. Two target sequences were simultaneously integrated into the vector to generate double tgRNA-pMgP237. Transgenic hairy roots were induced by *A. rhizogenes* ATCC15834 harboring double tgRNA-pMgP237 or the empty vector as a control. A total of nine target sequences on *LjCPR1* (**Supplementary Figure S2**) were selected using CRISPRdirect software (Naito et al., 2015).

**Figure 7:**
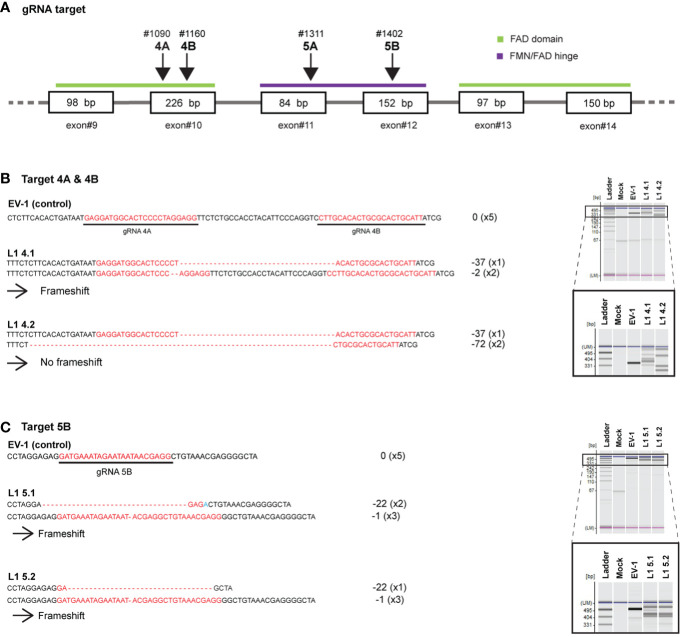
Disruption of *LjCPR1* gene in transgenic *Lotus japonicus* hairy roots by CRISPR/Cas9 system. Two sets of four gRNAs were designed to target *LjCPR1* gene in different domain regions **(A)**. The gRNA set nos. 4A and 4B **(B)** and 5B **(C)** successfully cut *LjCPR1* gene as confirmed by heteroduplex mobility assay (HMA) and sequencing, resulting in four *Ljcpr1*-KO hairy root mutant lines **(B, C)**.

Putative *Ljcpr1* hairy root mutant lines were selected using PCR and electrophoresis (HMA). Extra bands were observed in nine of the putative mutant hairy root lines but not in the control hairy root lines (**Supplementary Figure S3**), suggesting that mutations occurred in *LjCPR1* gene and produced heteroduplex PCR fragments. Genomic DNA fragments around the target sites were cloned and sequenced to confirm mutations. Mutated alleles were not found in the control lines, EV-1 (**Supplementary Figure S3**). No WT sequences were detected in all obtained mutants (**Supplementary Figure S3**). Four mutant hairy root lines with longer nucleotide deletions were chosen for further analysis (**Figure 7**). These mutant hairy root lines were generated by three gRNAs (gRNA 4A, 4B, and 5B) that successfully cut the target nucleotides on the FAD and FMN/FAD hinge domain of *LjCPR1* gene (**Figure 7A**), which resulted in three frameshift mutant lines (L1-4.1, L1-5.1, and L1-5.2) and one non-frameshift mutant line (L1-4.2) (**Figure 7B**).

Additionally, we analyzed the amino acid sequences of all *LjCPR1* mutant proteins from the mutant hairy root lines. All frameshift mutations in the hairy root mutant lines L1-4.1, L1-5.1, and L1-5.2 caused premature termination, resulting in a lower number of amino acids (approximately 200) when compared to those in the WT; however, the deletion of 72-bp allele in the non-frameshift mutant L1-4.2 did not cause early termination of the protein (**Supplementary File 1**). The non-frameshifting mutation resulted in the absence of amino acids 222nd to 246th in the WT *LjCPR1*. Structural analysis of the mutant protein indicated that the deletion of 72-bp nucleotides encoded one strand of α-helix near the FMN-binding domain (**Supplementary Figure S11A**). Nevertheless, comparing the WT and mutant LjCPR1 protein structure models using two different templates revealed that the loss of 24 amino acids in the *LjCPR1* mutant line L1-4.2 did not alter the overall conformation of the protein (**Supplementary Figures S11A, B**). The amino acid path in the mutant, where one α-helix was missing, was directly connected to amino acid chains and continued into a β-sheet structure with the same configuration as the WT *LjCPR1* (**Supplementary Figures S11A, B**).

**Figure 8:**
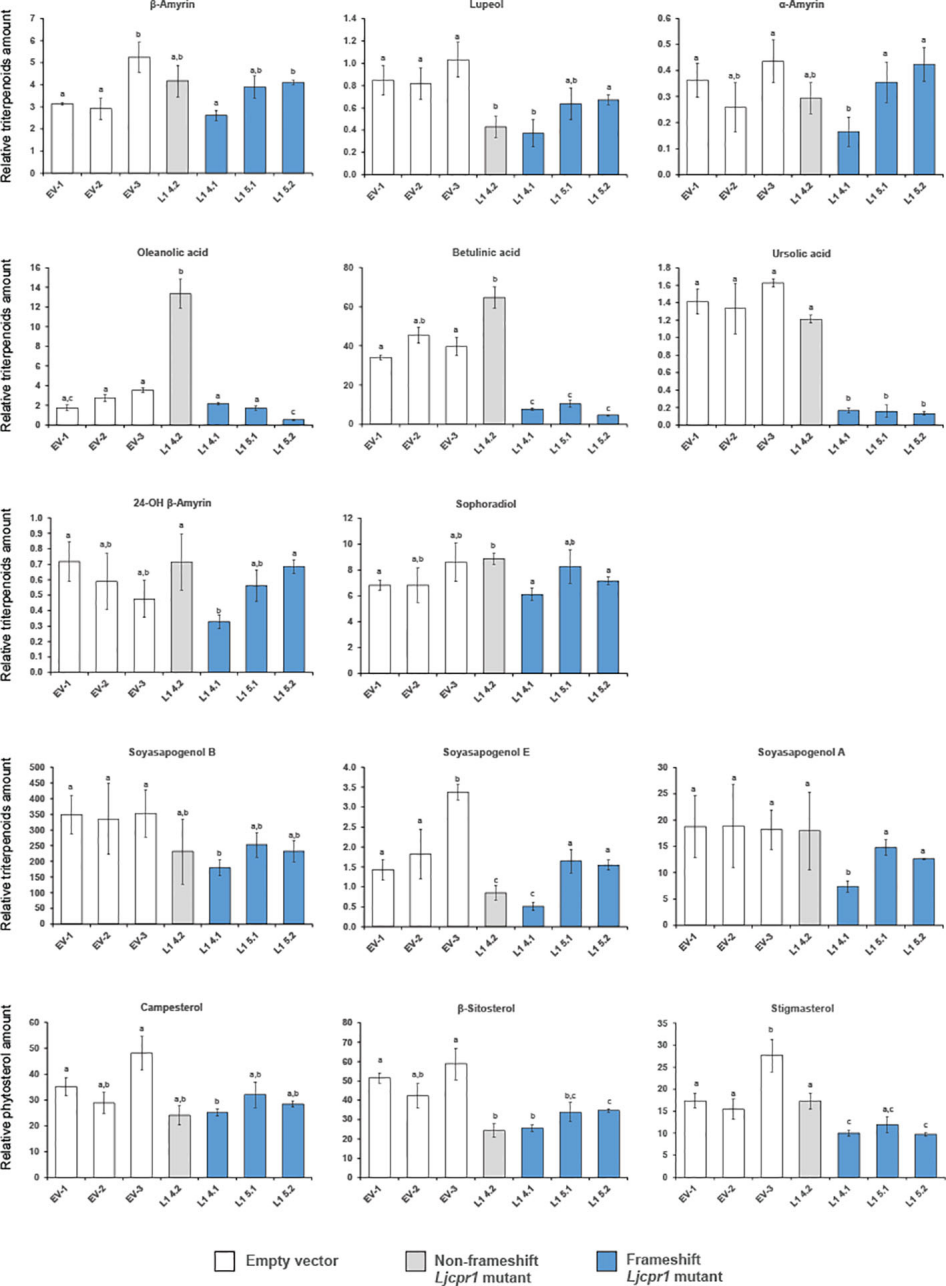
The relative triterpenoid and phytosterol contents of hairy root *Ljcpr-1* mutants analyzed using GC-MS. Relative triterpenoid and phytosterol amounts were normalized to those of asiatic acid as the internal standard and are presented as fold induction relative to the empty vector control. Data represent the mean of three technical replicates ± SD. Single-factor ANOVA was used for statistical comparisons. The different letters indicate significant differences (p < 0.05, one-way ANOVA followed by Tukey’s test). GC-MS, gas chromatography–mass spectrometry.

The triterpenoid profiles of control and *Ljcpr1* mutant hairy roots were then analyzed using GC-MS (**Figure 8**). All the frameshift mutants from different target regions showed significantly lower betulinic acid and ursolic acid content when compared to the control. Frameshift mutant lines L1-5.1 and L1-5.2 showed similar levels of lupeol, α-amyrin, and soyasapogenols compared to the control, while L1-4.1 showed significantly lower levels of those triterpenes compared to the control. All frameshift mutant lines showed similar levels of β-amyrin, 24-OH β-amyrin, and sophoradiol compared to the control. Interestingly, the non-frameshift L1-4.2 mutant line showed significantly higher oleanolic acid and betulinic acid than the control. In the phytosterol biosynthesis pathway, the knockout of *Ljcpr1* genes showed significantly lower β-sitosterol and stigmasterol amounts than the control. A similar experiment was repeated in another target region of *LjCPR1* gene to generate other *Ljcpr1*-KO hairy root mutant lines (**Supplementary Figure S12**). Similarly, the GC-MS result on these *Ljcpr1*-KO hairy root mutants showed significantly lower betulinic and ursolic acid levels and no effect on β-amyrin, 24-OH β-amyrin, sophoradiol, and soyasapogenol contents (**Supplementary Figure S12**). The total ion chromatogram (TIC) (**Supplementary Figure S13**) showed a significant decrease in the betulinic acid level of *Ljcpr1* knockout mutant hairy roots.

## 4 Discussion

Different structures of *LjCPR* genes were found in Miyakojima MG-20 and Gifu ecotype of *L. japonicus*. While both ecotypes of *L. japonicus* possess a single CPR class I gene and identical sequences, a minimum of two isoforms were found in LjCPR class II genes, branching into LjCPR2-1 and LjCPR2-2 clades in the phylogenetic tree. Only a single gene of each *LjCPR2-1* and *LjCPR2-2* gene were found in the Miyakojima MG-20 genome. However, multiple isoforms of *LjCPR2-1* and *LjCPR2-2* genes were found in the Gifu v1.2 genome. Interestingly, while Miyakojima MG-20 *LjmCPR2-1* showed an identical sequence to Gifu *LjgCPR2-1a*, *LjmCPR2-2* has no genes identical to any of the *LjgCPR2-2* isoforms. Based on expression analysis of *L. japonicus* Gifu v1.2 transcriptomic database, *LjCPR2-2* showed uniquely highest expression in immature flowers, compared to *LjCPR2-1* and *LjCPR1*. One of the main differences between the Miyakojima MG-20 and Gifu B-129 accessions is their flowering ability. Miyakojima MG-20 is known for its early and abundant flowering phenotypes, while Gifu B-129 is a late flowering phenotype with reduced flowering under fluorescent light. Therefore, the difference in the *LjCPR2-2* sequence between Miyakojima MG-20 and Gifu might contribute to explaining the phenotypic differences in the flowering ability of these ecotypes.

The MeJA treatment on *L. japonicus* hairy roots revealed segregation between LjCPR class I and II regulatory mechanisms. The results suggested that *LjCPR1* show regulatory mechanisms similar to betulinic acid biosynthetic genes *LUS* and *CYP716A51*, while *LjCPR2*s show a regulatory mechanism similar to soyasaponin biosynthetic genes *bAS*, *CYP93E1*, and *CYP72A61* regarding MeJA elicitation (**Figure 2**). However, even though *LUS* showed downregulated expression, the lupeol levels showed a significant increase of up to four times after 24 h when compared to control, similar to α-amyrin production (**Figure 4**). The increased lupeol and α-amyrin levels were possibly due to the accumulation of unconverted lupeol or α-amyrin into betulinic acid and ursolic acid, respectively, as the *CYP716A51* expression was very low. Interestingly, these triterpenoid biosynthetic pathways and CPR classes were supported by gene co-expression analysis showing that *LjCPR1* has a stronger correlation value with *CYP716A51* than *LjCPR2*, while *LjCPR2* has a stronger correlation value with *bAS*, *CYP93E1*, and *CYP72A61* than *LjCPR1*, as previously reported in Istiandari et al. (2021). The same correlation of LjCPR classes toward different CYPs is not a coincidence, suggesting that different CPR classes might have specific regulatory mechanisms with different CYPs involved in triterpenoid biosynthesis in *L. japonicus*.

*Ljcpr1* and *Ljcpr2-1 LORE-1* insertion mutants also exhibited different physiological changes in pod length and number. The loss of *LjCPR1* gene resulted in a significantly reduced pod length and number than the loss of *LjCPR2-1* gene. The significant involvement of *LjCPR1* gene in pod development might be related to the higher expression of *LjCPR1* in pods and seeds compared to *LjCPR2* (**Figure 1**). *LjCPR1* was also strongly correlated with adenylate translocator and abscisic acid (ABA) regulation (PCC value > 0.75, **Supplementary Table S8**). Adenylate translocator was reported to be responsible for translocating starch for accumulation in maize endosperms (Shannon et al., 1998) to nourish the embryo (Yan et al., 2014). ABA plays a very important role in seed development, dormancy, and germination (Xiong and Zhu, 2003). Loss of *LjCPR1* gene might compromise adenylate translocator function and ABA regulation in seed and pod development in *L. japonicus*. Furthermore, the fact that the loss of *LjCPR1*, but not *LjCPR2*, compromised pod number and length in *L. japonicus* also suggests that *LjCPR1* is crucial to support CYPs and other electron acceptors involved in seed development.

Previous gene co-expression analysis results (Istiandari et al., 2021) and MeJA treatment on *L. japonicus* hairy roots in this study showed that *LjCPR2*s had a strong correlation with *bAS*, CYP93E2, and *CYP72A61* genes. This notion was supported by the analysis of *Ljcpr2-1* loss-of-function mutants in this study that subsequently showed a significant reduction of β-amyrin, 24-OH β-amyrin, and sophoradiol when compared to *Ljcpr1* mutants and WT. However, it was previously suggested that *LjCPR1* had a strong correlation with *CYP716A51* (Istiandari et al., 2021), which was shown by the significant reduction in betulinic acid and ursolic acid levels in *Ljcpr1* loss-of-function mutants. However, betulinic acid and ursolic acid were also reduced in *Ljcpr2-1* mutants, suggesting that *LjCPR1* is not acting alone in supporting *CYP716A51*. There might be synergistic work of *LjCPR1* and *LjCPR2-1* in supporting *CYP716A51*. However, in the case of *CYP93E1* and *CYP72A61*, the vital role of *LjCPR2-1* cannot be complemented by the presence of *LjCPR1*. Also interestingly, whereas both *Ljcpr1* and *Ljcpr2-1* mutants showed reductions in β-sitosterol content, only *Ljcpr1* mutants showed a lower level of stigmasterol in hydroponic-cultured mutants (**Supplementary Figure S10**). In *A. thaliana*, stigmasterol is synthesized from β-sitosterol by CYP710A, while campesterol and β-sitosterol biosynthesis do not involve CYPs (Morikawa et al., 2006). Therefore, the involvement of CYP710A in the stigmasterol biosynthesis pathway might be correlated stronger with *LjCPR1* than with *LjCPR2*.

Gene editing using CRISPR/Cas9 confirmed the function of a specific CPR class. *Ljcpr1*-KO mutant hairy roots exhibited a varied triterpenoid profile. The premature termination of the frameshift mutations resulted in a shorter *LjCPR1* (approximately 200 amino acids less) than the WT, with the missing 200 amino acids encoding the conserved domain critical for CPR activity, including FAD- and NADPH-binding sites. Consequently, the activity of CYP716A51, which was involved in the conversion of β-amyrin into oleanolic, betulinic, and ursolic acids, significantly reduced (**Figure 8**). Similar to the *LORE1* loss-of-function mutant line *Ljcpr1*, the knockout of *Ljcpr1* gene reduced the betulinic acid and ursolic acid contents, while β-amyrin, 24-OH β-amyrin, and sophoradiol contents remained unchanged in all mutants.

In contrast, the non-frameshifting mutant line, L1-4.2, displayed a triterpenoid level similar to the WT. The preserved configuration of the native protein in the mutant *LjCPR1* may allow it to function similarly to the protein of the WT (**Supplementary Figures S11A, B**). Metabolite profile analysis revealed differences in the production levels of lupeol, oleanolic acid, and soyasapogenol E in the *Ljcpr1*-KO hairy root mutant line L1-4.2. The absence of α-helix in the mutant *LjCPR1* may have altered specific protein–protein interactions with CYPs responsible for triterpenoid production, affecting electron transfer rates and CYP activity. Further studies, such as those producing recombinant CPR and conducting *in vitro* assays, are needed to confirm the impact of CPR mutations on CYP activity.

Although the loss of some amino acids may disrupt protein conformation, non-frameshift protein mutants have a higher probability of retaining their functions than frameshift or nonsense mutations. In a study by Bermejo-Das-Neves et al. (2014) on non-frameshifting indels in human genetic variation in Mendelian disease, out of 2,163 NFS-Indels, 757 were disease-causing variants, and 1,406 were neutral or unknown. These data suggest that the mutant *LjCPR1* from the hairy root mutant line L1-4.2 may be a neutral non-frameshifting mutant. Changes in the amino acid sequence also raise the possibility of altering the CYP : CPR binding motifs (Istiandari et al., 2021), suggesting that these non-functional LjCPR1 mutant proteins may still bind to CYPs or be replaced by other redox proteins, such as *LjCPR2s* or cytochrome *b*_5_, due to changes in protein–protein affinity (Esteves et al., 2020).

While each mutant displayed differences in triterpenoid profiles, the result of *Ljcpr1*-KO mutant hairy roots confirmed the findings in *Ljcpr1* loss-of-function *LORE1* insertion mutant plants (**Figure 6**). These findings highlight the significant involvement of *LjCPR1* in betulinic acid and ursolic acid production while highlighting that *LjCPR1* is not essential for producing β-amyrin, oleanolic acid, 24-OH β-amyrin, and sophoradiol. However, our study only obtained *Ljcpr1*-KO hairy root mutants, and further research is needed to confirm the function of *LjCPR2*s in *L. japonicus*. Complementation assays of *LjCPR2-1* or the generation of *LjCPR2-1* overexpression lines should be performed to confirm the critical role of *LjCPR2-1* in the production of β-amyrin, oleanolic acid, 24-OH β-amyrin, sophoradiol, and soyasapogenols *in planta*. The unaffected oleanolic acid content in the *LORE1 Ljcpr1* mutants raises questions about the involvement of *LjCPR2s* in *bAS* and CYP716A51, which appear to be unaffected by the loss of *Ljcpr1* gene, unlike the effect on betulinic acid and ursolic acid biosynthesis.

The level of oleanolic acid was not changed in the *LORE1 Ljcpr1* mutants, which might be due to the high level of β-amyrin in *Ljcpr1* mutants compared to that of the *Ljcpr2-1* mutants. However, it is known that CPR can only support CYPs, not OSCs. Therefore, this result implies that there might be a more complex regulatory mechanism for how CPR works with CYPs. There might be a possibility that CYP716A51 can make a specific complex with different OSCs (bAS, aAS, and LUS) and create a specific metabolon. Metabolons are temporary multi-protein complexes of sequential enzymes that mediate substrate channeling (Zhang and Fernie, 2021). Metabolons have been found in several primary metabolisms, such as monolignol biosynthesis in *Populus trichocarpa* (Lin et al., 2021), or secondary metabolisms, such as camalexin biosynthesis in *A. thaliana* (Mucha et al., 2019) and dhurrin biosynthesis in *Sorghum bicolor* (Nielsen et al., 2008), which involved membrane-bound CYPs, CPRs, and other enzymes. The significant reduction of betulinic acid and ursolic acid but not in oleanolic acid level suggested that LjCPR1 is specifically involved in the betulinic acid and/or ursolic acid biosynthetic pathways, but not oleanolic acid biosynthesis. Instead, it was suggested that LjCPR2-1 might be involved more closely in the oleanolic acid biosynthetic pathway with bAS as the first enzyme converting 2,3-oxidosqualene into β-amyrin as a substrate for CYP716A51. However, more studies are needed to confirm this hypothesis. Protein–protein interaction analysis using techniques such as bimolecular fluorescence complementation or protoplast two-hybrid assay should be conducted to reveal the structural proteins of the metabolon scaffold *in planta* (Nielsen et al., 2008; Mucha et al., 2019).

Both CPR classes were reported to be able to support the *in vitro* activities of CYPs involved in specialized metabolism (Rana et al., 2013). However, based on numerous functional analyses *in planta*, CPR class I was believed to be responsible for basal or constitutive metabolisms, while CPR class II was more responsible for adaptation and defense mechanisms, involving numerous specialized metabolisms (Rana et al., 2013; Parage et al., 2016; Huang et al., 2021; Zou et al., 2021). This study showed that LjCPR1 is closely involved with CYP716A51, a C-28 oxidase, which is involved in triterpenoid biosynthesis, one of the specialized metabolisms in *L. japonicus*. Based on the elicitor treatment, the LjCPR class I showed no induction (**Figure 3**), which is in line with the previous notion that only CPR class II is inducible (**Figure 3**). The result that *CYP716A51* was not inducible during methyl jasmonate treatment (**Figure 3**) might suggest a function for C-28 oxidized triterpenes in *L. japonicus* other than as a defense mechanism. In line with phenotypic change observed in the seeds of *Ljcpr1* mutant plants (**Figure 5**) and expression level changes in mature seeds (**Figure 1**), LjCPR1 and CYP716A51 might also have physiological roles in the seed development of *L. japonicus*. To validate this hypothesis, further analysis should be conducted on the expression of the β-glucuronidase (GUS) reporter gene driven by the native promoters of *LjCPR1* and *LjCPR2* in response to MeJA.

In *Medicago truncatula*, CYP716A12 is involved in oleanane-derived hemolytic sapogenin biosynthesis. During the developmental stages, such as the reproductive phase, *CYP716A12* showed a significant increase in expression level and an increase in hemolytic sapogenin content. Ten-week-old *M. truncatula cyp716a12* mutant plants showed dwarf phenotype compared to WT. This finding suggested a possible dual role of hemolytic sapogenins in defense and plant developmental growth in *M. truncatula* (Carelli et al., 2011). Functional analysis and phenotypic observation of *cyp716a51* mutant of *L. japonicus* might provide insight into LjCPR1 and CYP716A51 involvement in plant primary metabolism.

## Data availability statement

The datasets presented in this study can be found in online repositories. The names of the repository/repositories and accession number(s) can be found in the article/**Supplementary Material**.

## Author contributions

PI, EF, and TM designed the experiments. PI conducted all of the experiments, analyzed the results, and wrote the whole manuscript. EF, SY, HS, and TM conceived and supervised the study. SY, HS, and TM made the manuscript revisions. All authors contributed to the article and approved the submitted version.
