# Supplementary material for: Class I and II NADPH-cytochrome P450 reductases exhibit different roles in triterpenoid biosynthesis in Lotus japonicus
Source: Front Plant Sci. 2023 Aug 9;14:1214602. doi: 10.3389/fpls.2023.1214602 (PMC10445947; doi:10.3389/fpls.2023.1214602)
Supplement: Supplementary file 1 [file DataSheet_1.pdf]

Supplementary Materials

Supplementary Figures

Figure S1. PCR genotyping of *LORE1* insertion *Ljcpr* mutant.

Figure S2. The gRNA design targeting *LjCPR1* gene.

Figure S3. Disruption of the *LjCPR1* gene in transgenic *L. japonicus* hairy roots by CRISPR/Cas9 system using gRNA target 2B.

Figure S4. Mass spectra of target compounds and authentic standards used to confirm triterpenoids peak in GC-MS chromatogram of *L. japonicus* hairy root and plant root.

Figure S5. Location of *LjCPR* genes in the genome of (A-B) Miyakojima MG-20 and (C-D) Gifu B-129 ecotype.

Figure S6. Molecular phylogenetic tree of CPR class I and II from different plant species.

Figure S7. Multiple sequence alignment of LjCPR amino acid sequences from Miyakojima MG-20 and Gifu B-129 ecotype.

Figure S8. TIC chromatogram of hairy root extracts after 0, 12, 24, and 48 h of MeJA treatment.

Figure S9. Mass spectra of phytosterols compared to NIST library compared to standard compound mass spectrum obtained from previous experiment.

Figure S10. The relative amount of triterpenoids and phytosterol content of hydroponic-cultured *L. japonicus LORE1* insertion mutant roots analyzed by GC-MS.

Figure S11. Crystal structure modelling of wild-type LjCPR1 overlapped with non-frameshift mutant LjCPR1 from *Ljcpr1*-KO mutant hairy root line L1-4.2 using 5gxu.1.A and A0A0R4J338.1.A as template.

Figure S12. The relative amount of A) triterpenoids and B) phytosterol content hairy root *Ljcpr-1* (target 2B) mutants analyzed by GC-MS.

Figure S13. TIC scan of GC-MS chromatogram of *Ljcpr1* knockout hairy root mutants.

Supplementary Tables

Table S1. List of accession numbers of CPR genes and amino acid sequences from other plant species used for phylogenetic analysis in this study

Table S2. (A) Amino acid and (B) nucleotide sequence identity matrix of LjCPRs from Miyakojima MG-20 and Gifu B-129 ecotype.

Table S3. Primer sequences used for qPCR analysis of MeJA-treated *L. japonicus* hairy roots

Table S4. All *LORE1* insertions in the genome of the selected *Ljcpr1* and *Ljcpr2-1* mutant lines (lotus.au.dk)

Table S5. *LORE1* genotyping primer sequences

Table S6. Primer sequences used to construct gRNAs targeting *LjCPR1* gene

Table S7. List of m/z values for the target ion and qualifier ion used in GC-MS analysis

Table S8. Co-expression analysis of closely correlated genes with CPR class I and II in *L. japonicus*

Supplementary Files

File 1. Amino acid sequences of mutant LjCPR1 in *Ljcpr1-KO* mutant in hairy roots

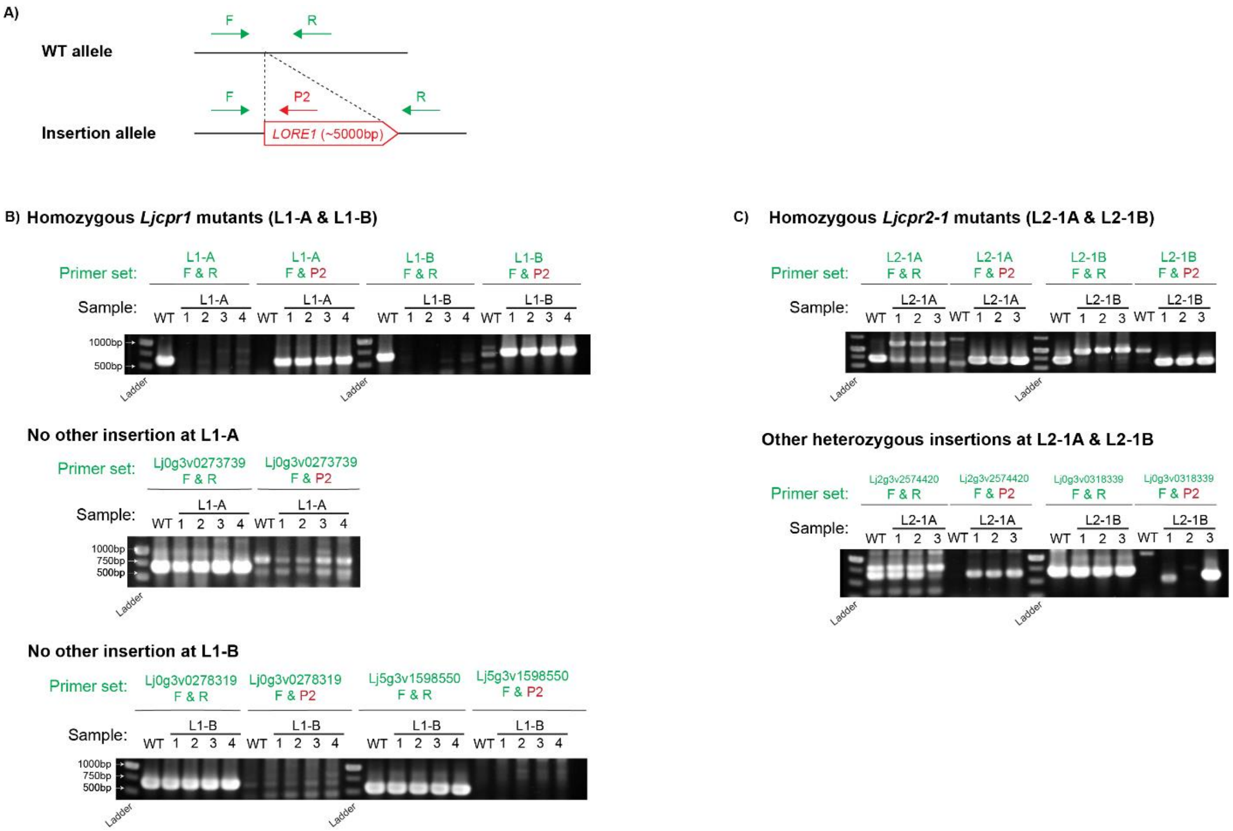

Figure S1. PCR genotyping of *LORE1* insertion *Ljcpr* mutant. A) Primer pairs for *LORE1* PCR genotyping. B) Gel electrophoresis of soil-cultured *Ljcpr1 LORE1* insertion mutants confirmed single insertion homozygous L1-A and L1-B *LORE1* insertion mutation and no other *LORE1* exonic insertion present in other expected genes. C) Gel electrophoresis of soil-cultured *Ljcpr2-1 LORE1* insertion mutants confirmed non-single insertion homozygous L2-1A and L2-1B *LORE1* insertion mutation with heterozygous *LORE1* exonic insertion presents in other expected genes. Hydroponic-cultured mutant seeds were collected from these homozygous mutant plants and also confirmed by PCR as described above. Minimum three homozygous mutant plants per lines were obtained.

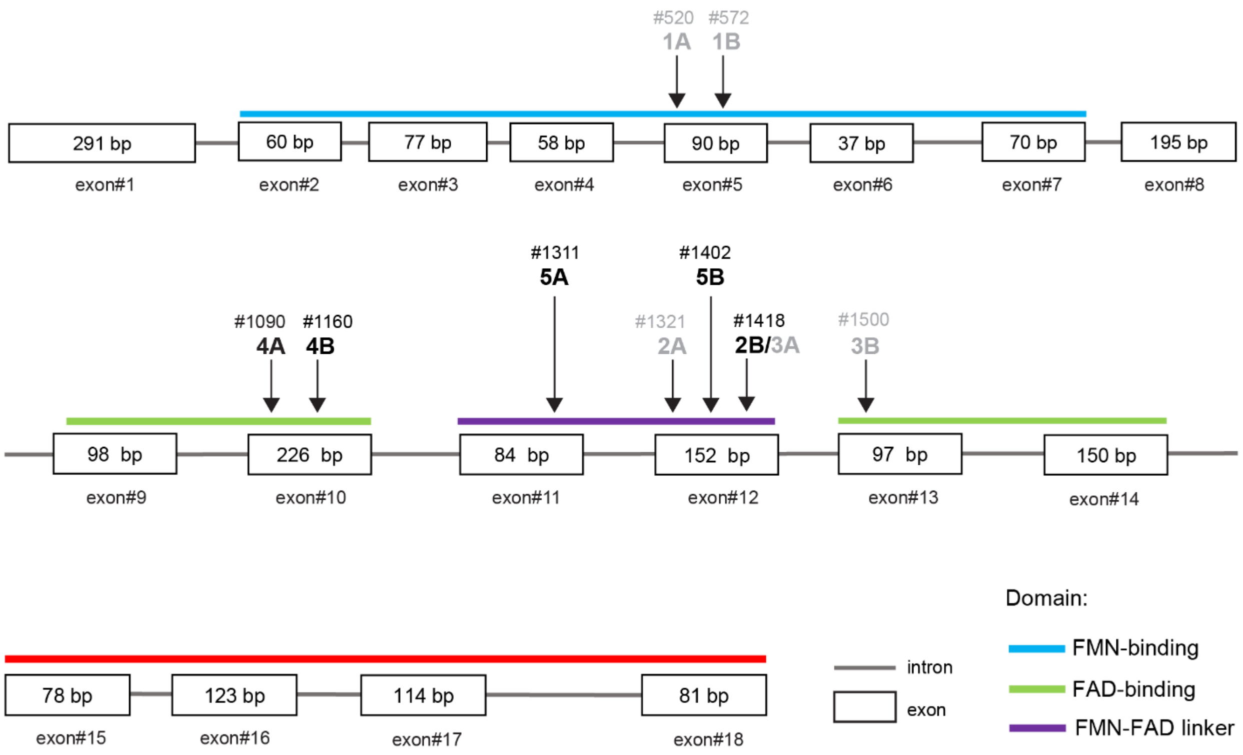

Figure S2. The gRNA design targeting *LjCPR1* gene. Five sets of duplex-gRNA targeting LjCPR1 gene were designed based on https://crispr.dbcls.jp/. The number in hashtag represents the base pair position of the target gRNA sequence in the exon part. The target number with grey color represents the target which Cas9 failed to disrupt (no deletions observed).

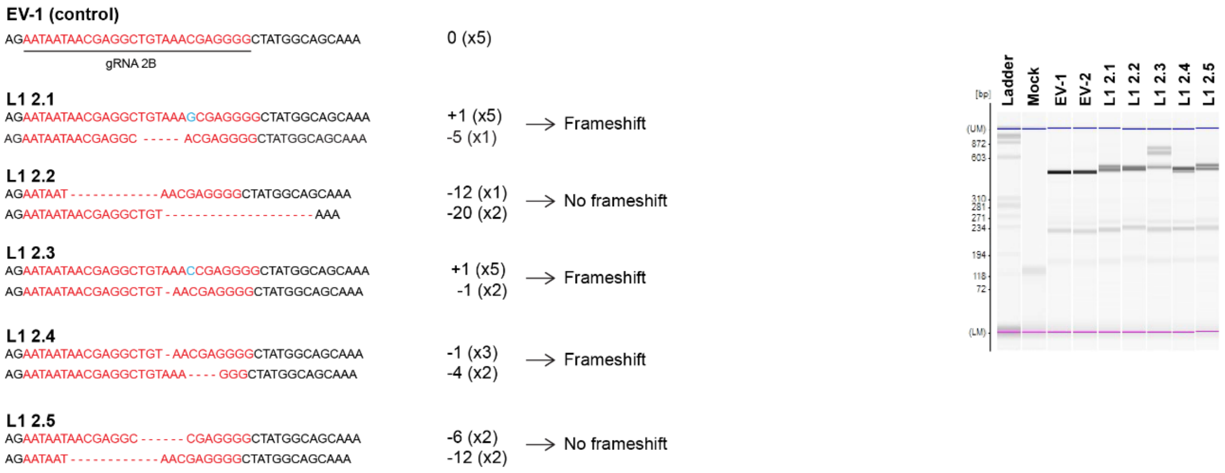

Figure S3. Disruption of the *LjCPR1* gene in transgenic *L. japonicus* hairy roots by CRISPR/Cas9 system using gRNA target 2B.

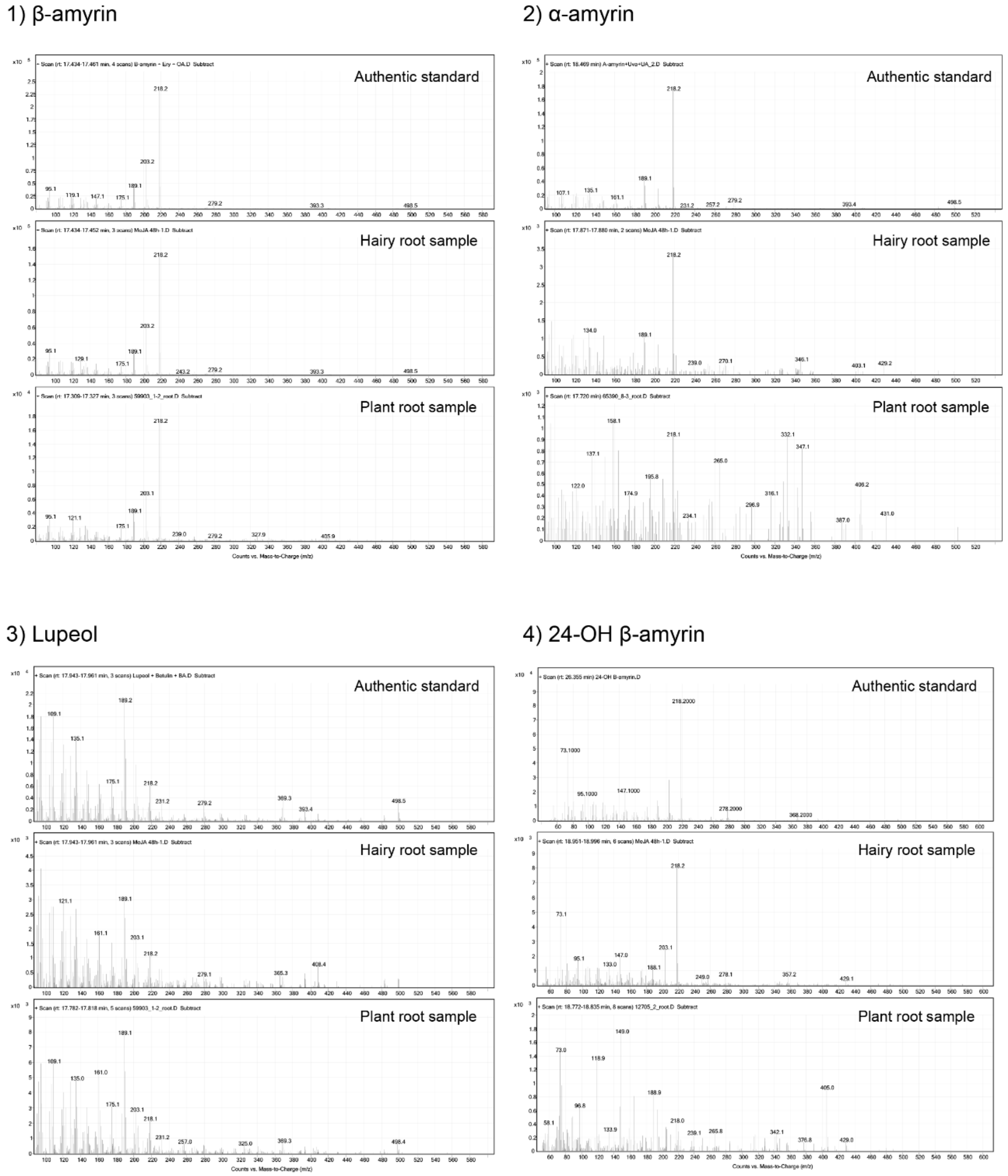

Figure S4. Mass spectra of target compounds and authentic standards used to confirm triterpenoids peak in GC-MS chromatogram of *L. japonicus* hairy root and plant root. Number indicates the order of metabolites as mentioned in Figure 2.

8) Oleanolic acid

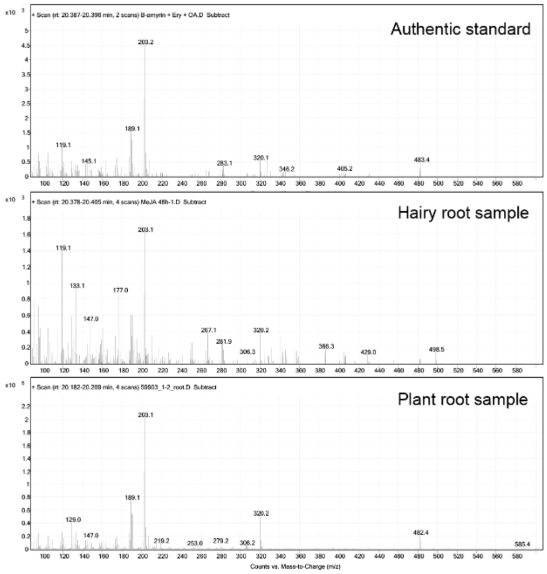

9) Betulinic acid

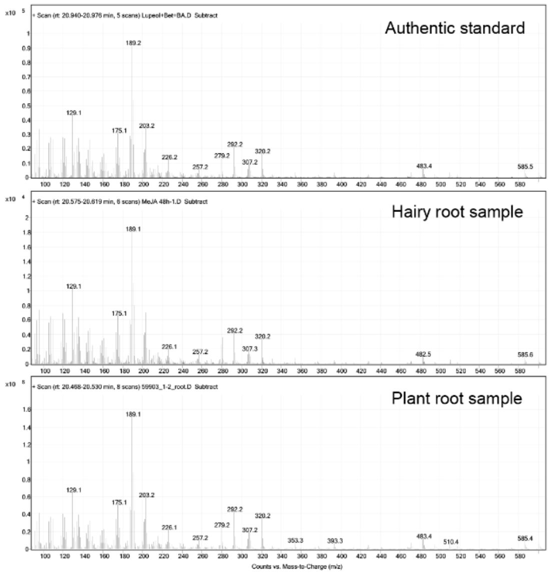

10) Sophoradiol

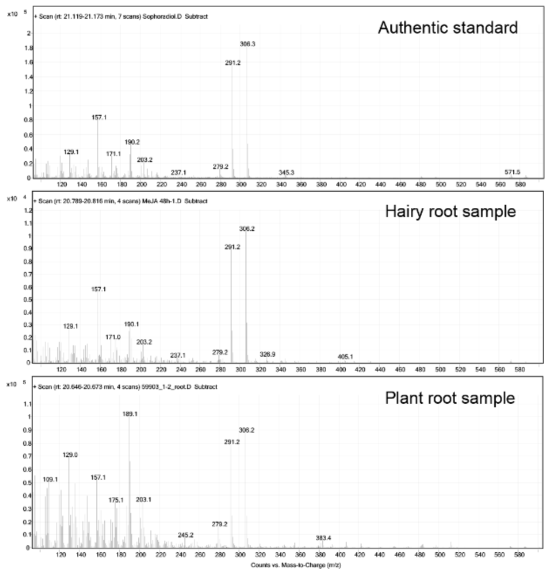

11) Ursolic acid

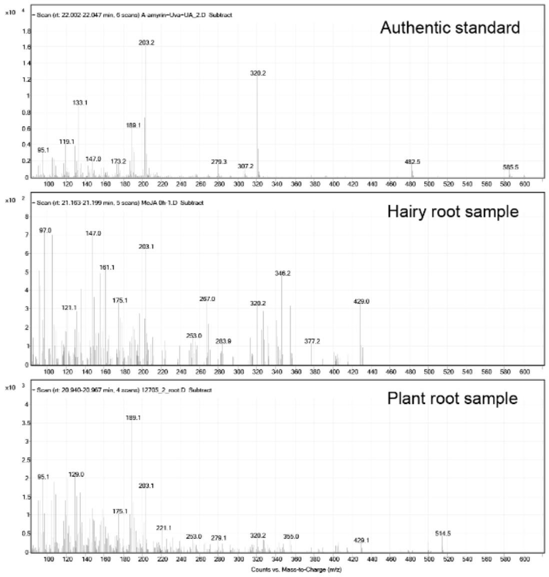

Figure S4. Cont.

12) Soyasapogenol E

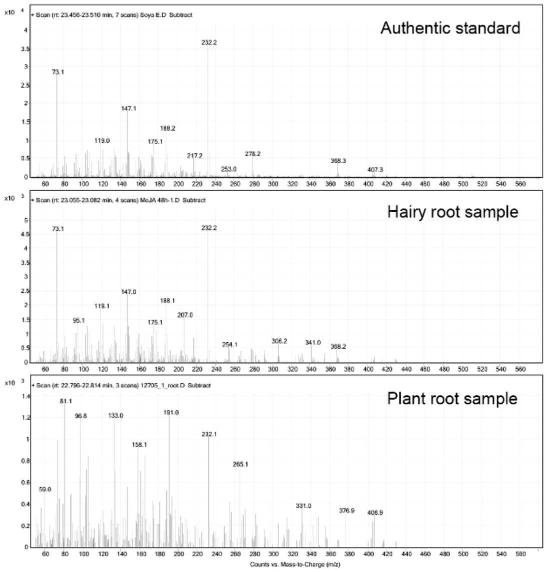

13) Soyasapogenol B

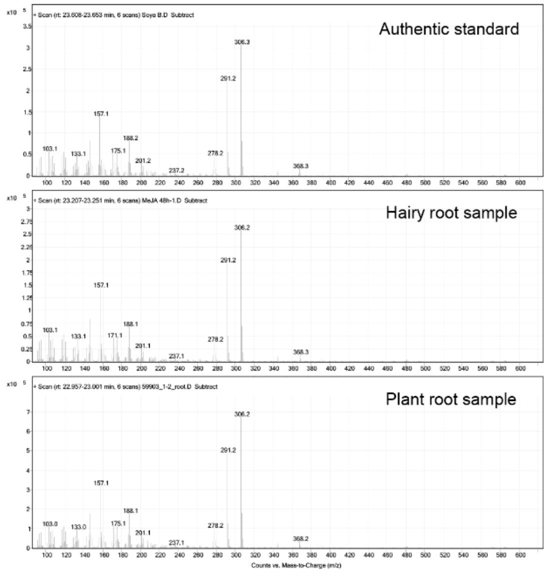

14) Soyasapogenol A

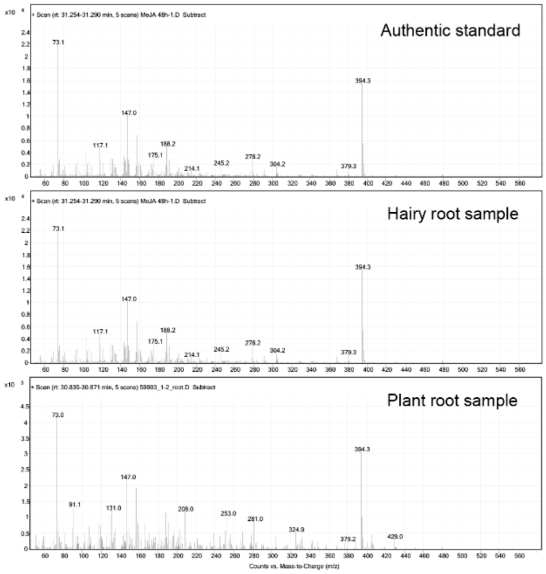

18) Asiatic Acid

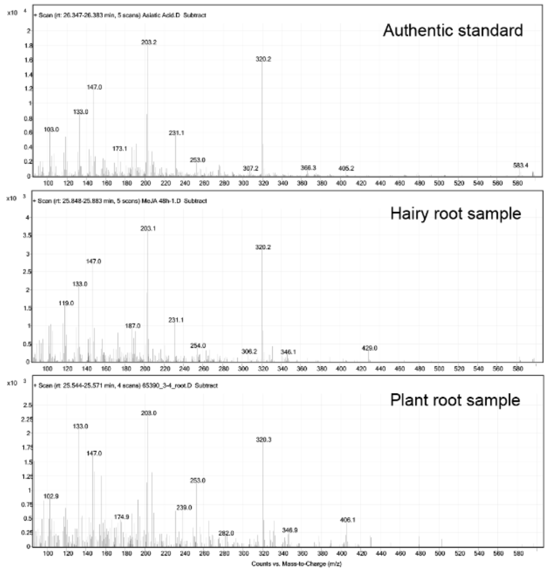

Figure S4. Cont.

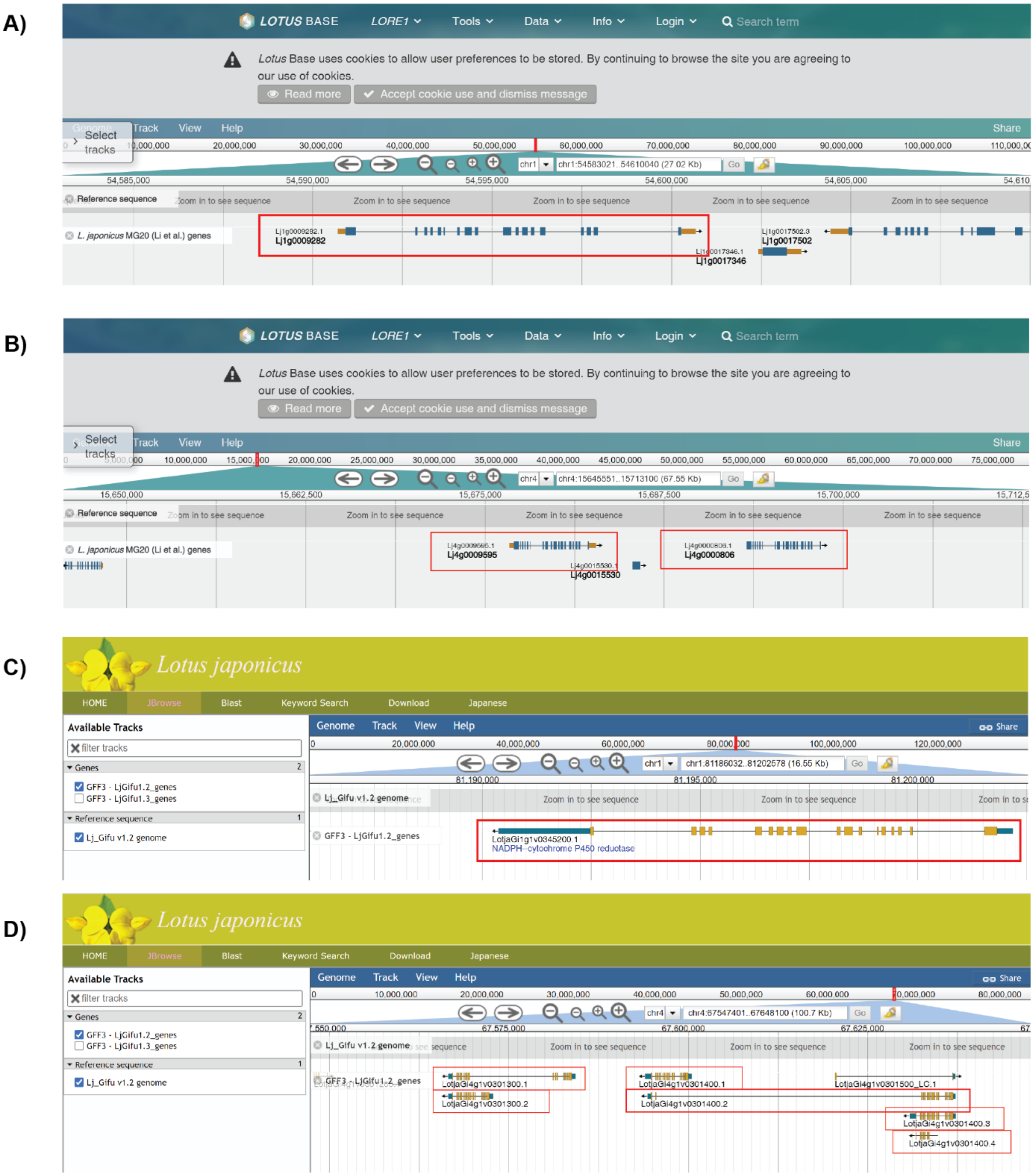

Figure S5. Location of LjCPR genes in the genome of (A-B) Miyakojima MG-20 and (C-D) Gifu B-129 ecotype. (A) and (C) are LjCPR class I genes in the genome of Miyakojima and Gifu, respectively. (B) and (D) are LjCPR class II genes in the genome of Miyakojima and Gifu, respectively. *Lotus japonicus* Miyakojima and Gifu ecotype genome information was obtained from https://lotus.au.dk/ and http://viewer.shigen.info/lotus, respectively.

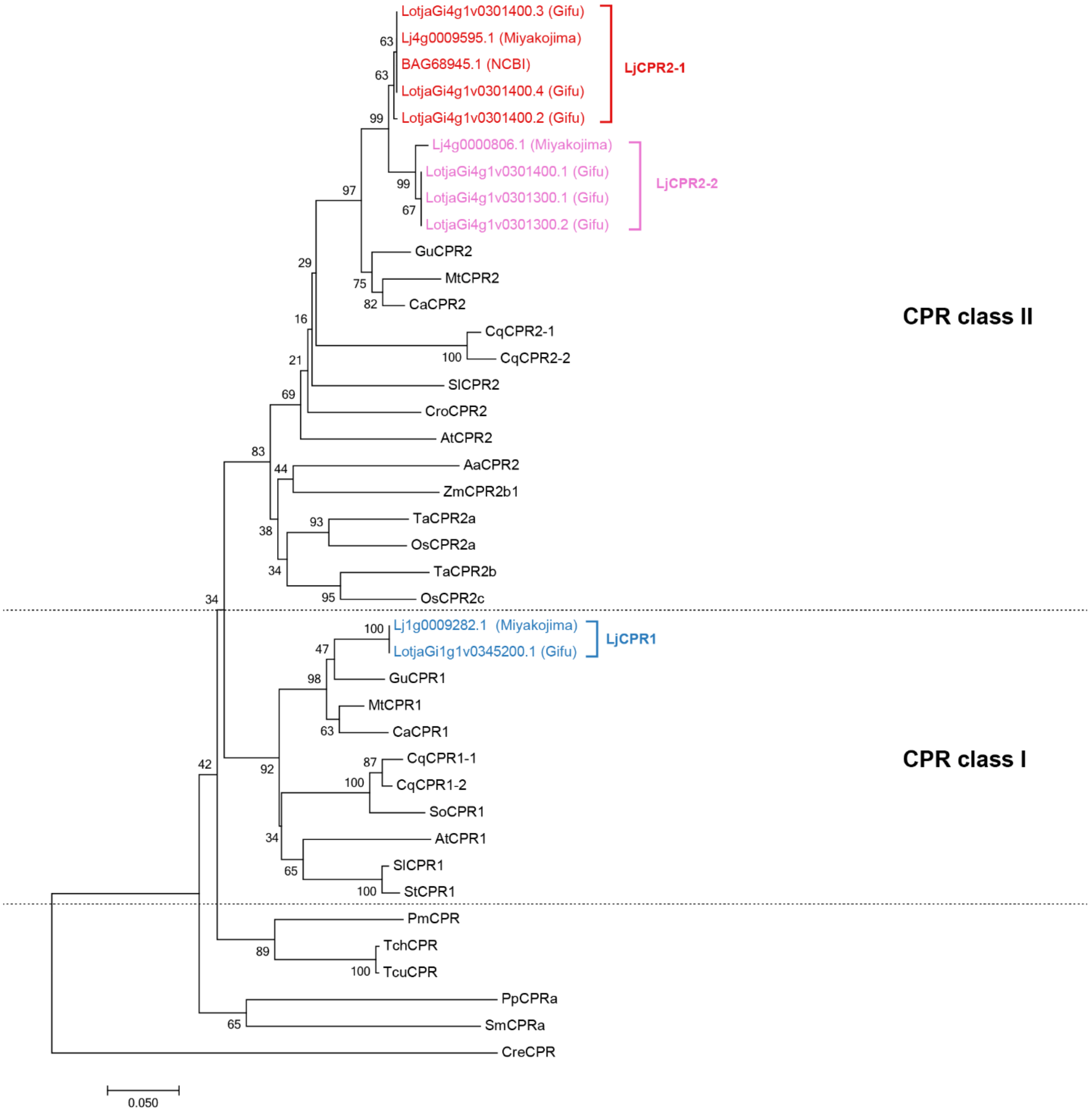

Figure S6. Molecular phylogenetic tree of CPR class I and II from different plant species. *L. japonicus* CPR genes and gene ID from Miyakojima (MG20) and Gifu ecotype are indicated in colored text. LjCPR2-1, LjCPR2-2, and LjCPR1 are indicated in red, pink, and blue color, respectively. CPR, cytochrome P450 reductase; Aa, *Artemisia annua*; At, *Arabidopsis thaliana*; Ca, *Cicer arietinum*; Cre, *Chlamydomonas reinhardtii*; Cro, *Catharantus roseus*; Cq, *Chenopodium quinoa*; Gu, *Glycyrrhiza uralensis*; Lj, *Lotus japonicus*; Mt, *Medicago truncatula*; Os, *Oryza sativa*; Pm, *Pseudotsuga menziesii*; Pp, *Physcomitrella patens*; Sl, *Solanum lycopersicum*; Sm, *Selaginella moellendorffii*; St, *Solanum tuberosum*; Ta, *Triticum aestivum*; Tch, *Taxus chinensis*; Tcu, *Taxus cupidata*; Zm, *Zea mays.*

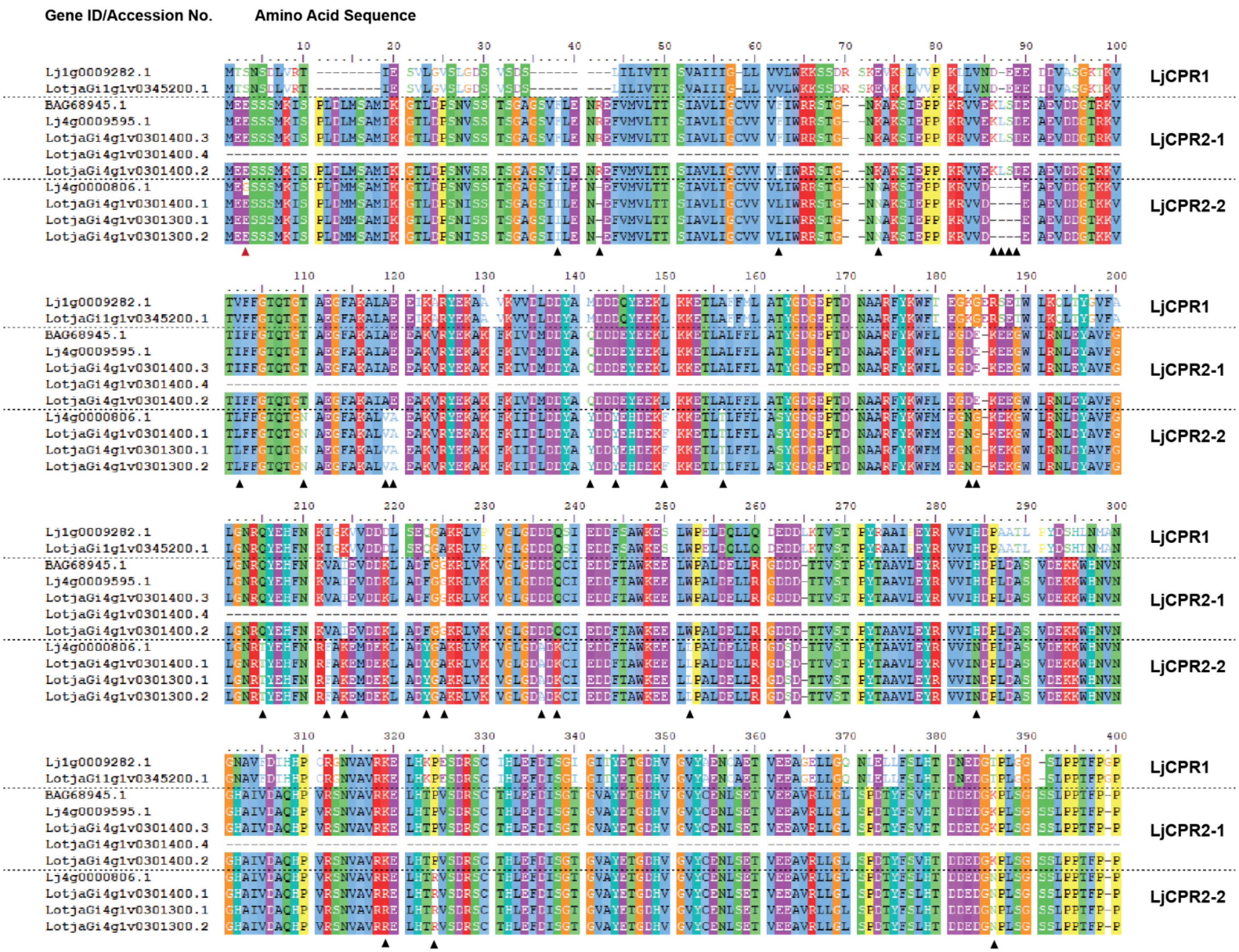

Gene ID/Accession No.
Amino Acid Sequence
Lj1g0009282.1
LotjaGi1g1v0345200.1
BAG68945.1
Lj4g0009595.1
LotjaGi4g1v0301400.3
LotjaGi4g1v0301400.4
LotjaGi4g1v0301400.2
Lj4g0000806.1
LotjaGi4g1v0301400.1
LotjaGi4g1v0301300.1
LotjaGi4g1v0301300.2
LjCPR1
LjCPR2-1
LjCPR2-2

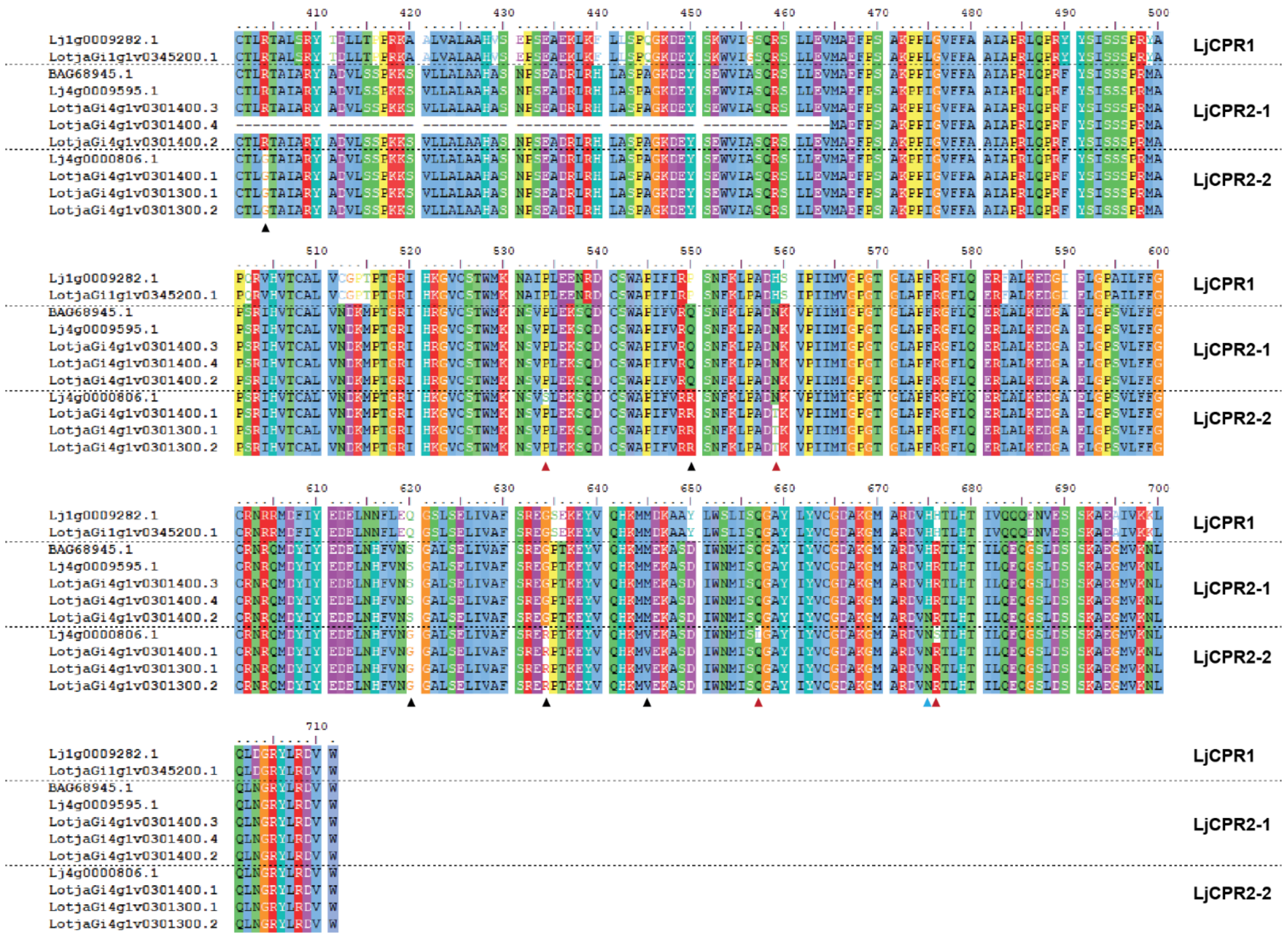

Figure S7. Multiple sequence alignment of LjCPR amino acid sequences from Miyakojima MG-20 and Gifu B-129 ecotype. The gene IDs and CPR sequences for Miyakojima (MG20) and Gifu ecotype were obtained from *Lotus japonicus* MG20 genome (Li et al., 2020) and Gifu v1.2 genome version, respectively. The block color represents more than 30% similarity among the sequences. Black triangle represents different amino acids between LjCPR2-1 and LjCPR2-2. Red triangle represents different amino acids between LjCPR2-2 from MG20 and Gifu ecotype. Blue triangle represents single amino acid difference between LjCPR2-1a and LjCPR2-1c from Gifu ecotype.

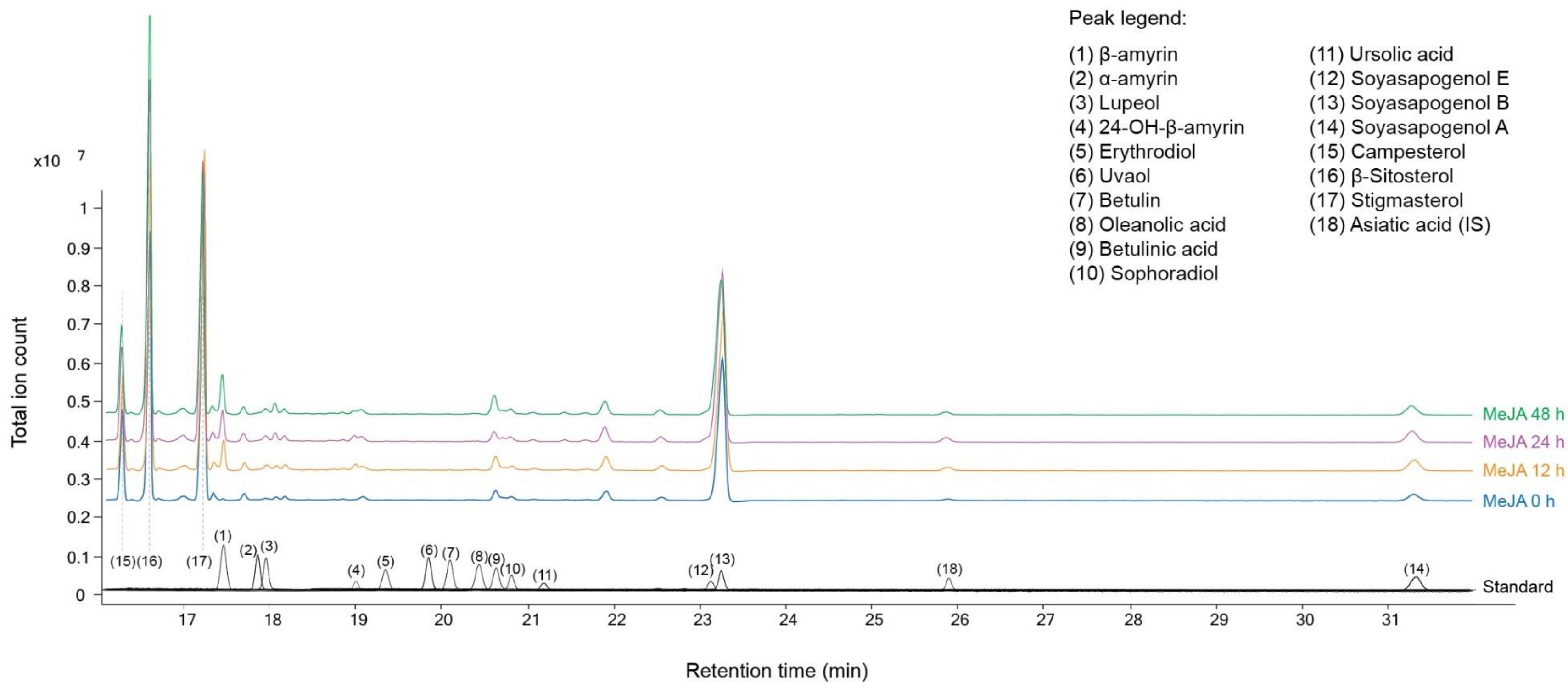

Figure S8. TIC chromatogram of hairy root extracts after 0, 12, 24, and 48 h of MeJA treatment. Peak number 1-14 are triterpenoids confirmed by standard compounds. Peak number 15-17 are phytosterols confirmed by NIST mass spectra (Fig. S4). Peak number 18 belongs to asiatic acid as internal standard confirmed by standard compound.

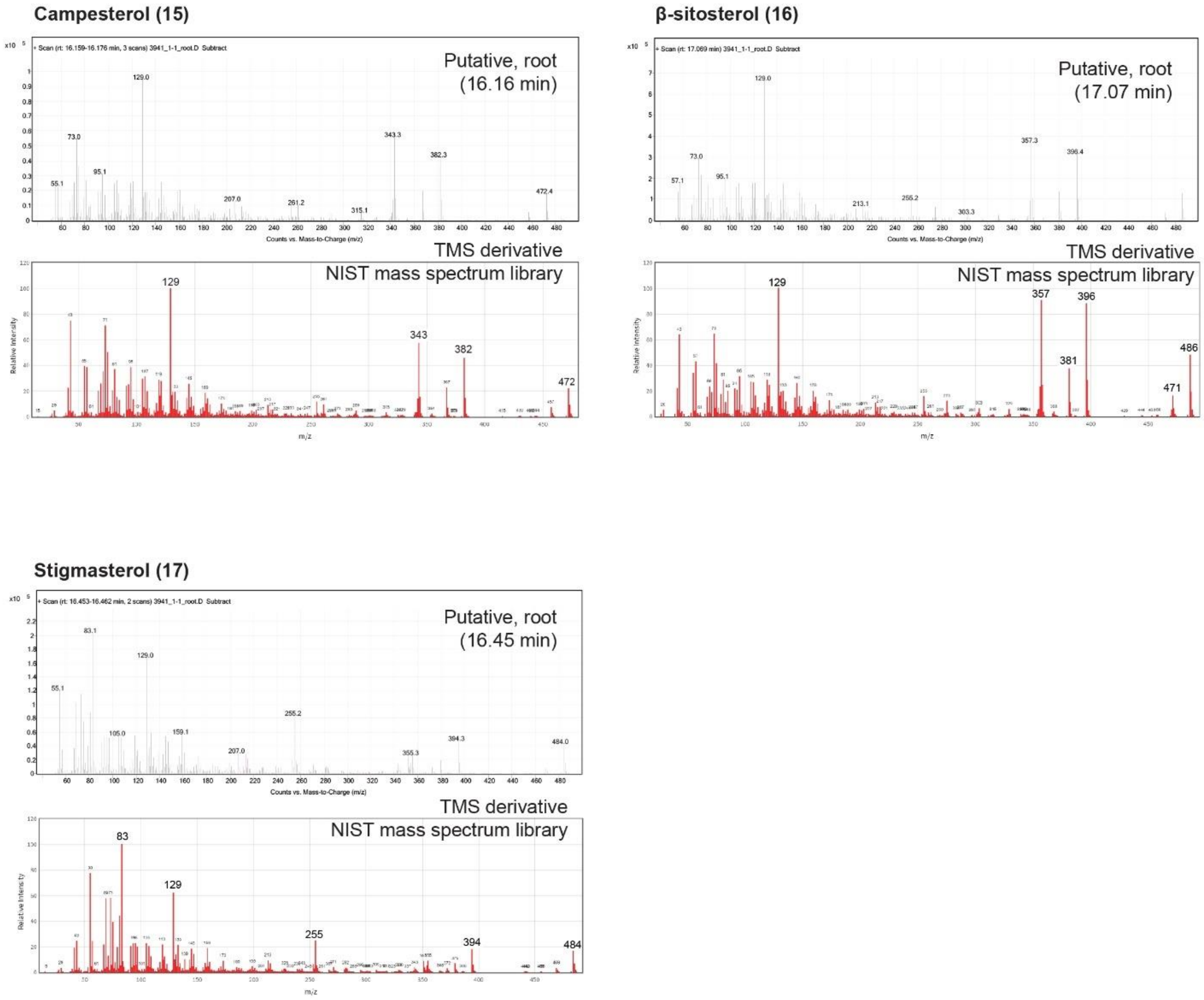

Figure S9. Mass spectra of phytosterols compared to NIST library compared to standard compound mass spectrum obtained from previous experiment.

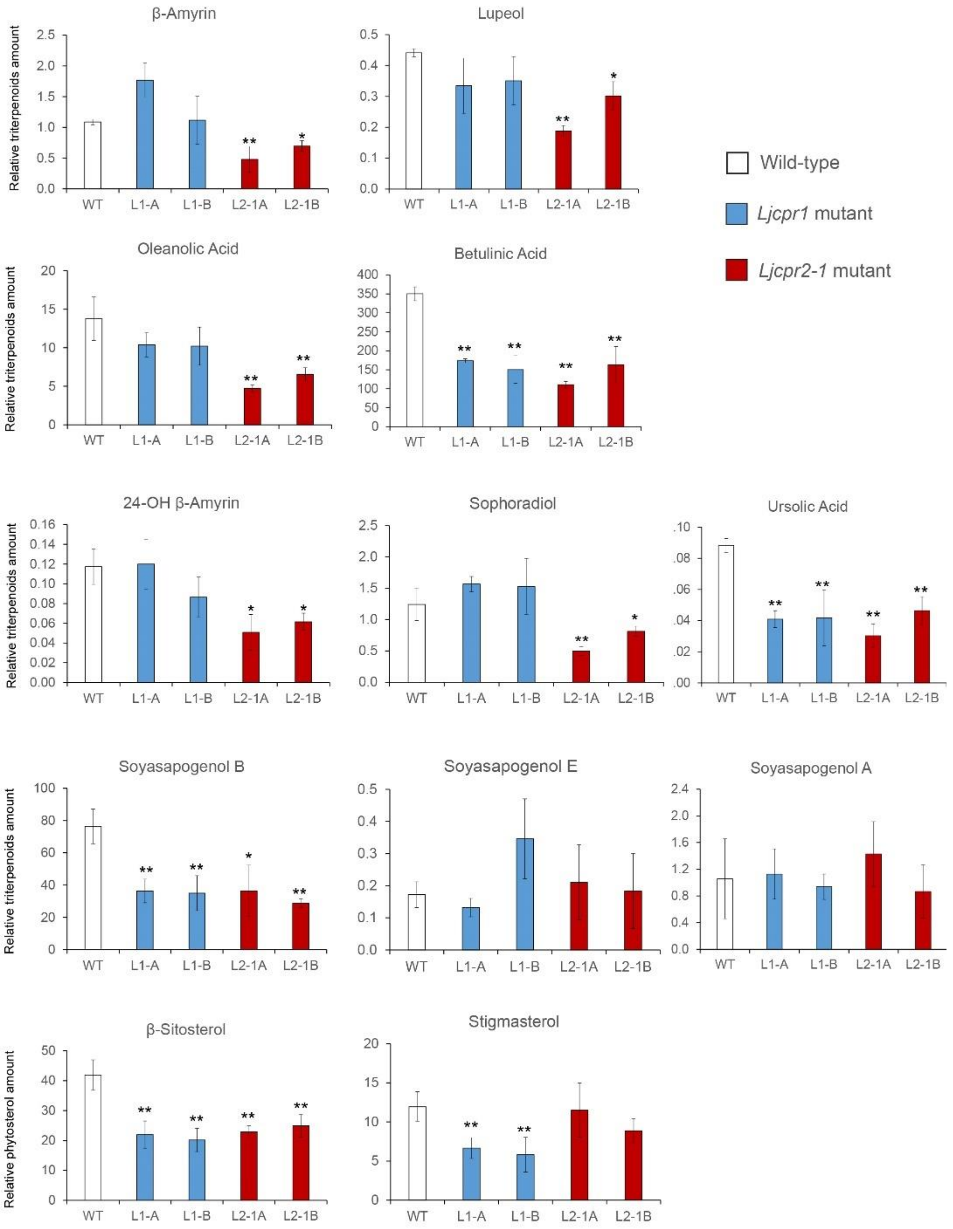

Figure S10. The relative amount of triterpenoids and phytosterol content of hydroponic-cultured *L. japonicus LORE1* insertion mutant roots analyzed by GC-MS. Relative triterpenoids and phytosterol amount were normalized to that of asiatic acid as internal standard and are presented as fold induction relative to the wild-type control (WT). Data represent the mean of three biological replicates ± SD. Single-factor ANOVA with Tukey's post-hoc test was used for statistical comparison to wild-type control (WT). Values were considered statistically significant at *P<0.05 and **P<0.01. SD, standard deviation.

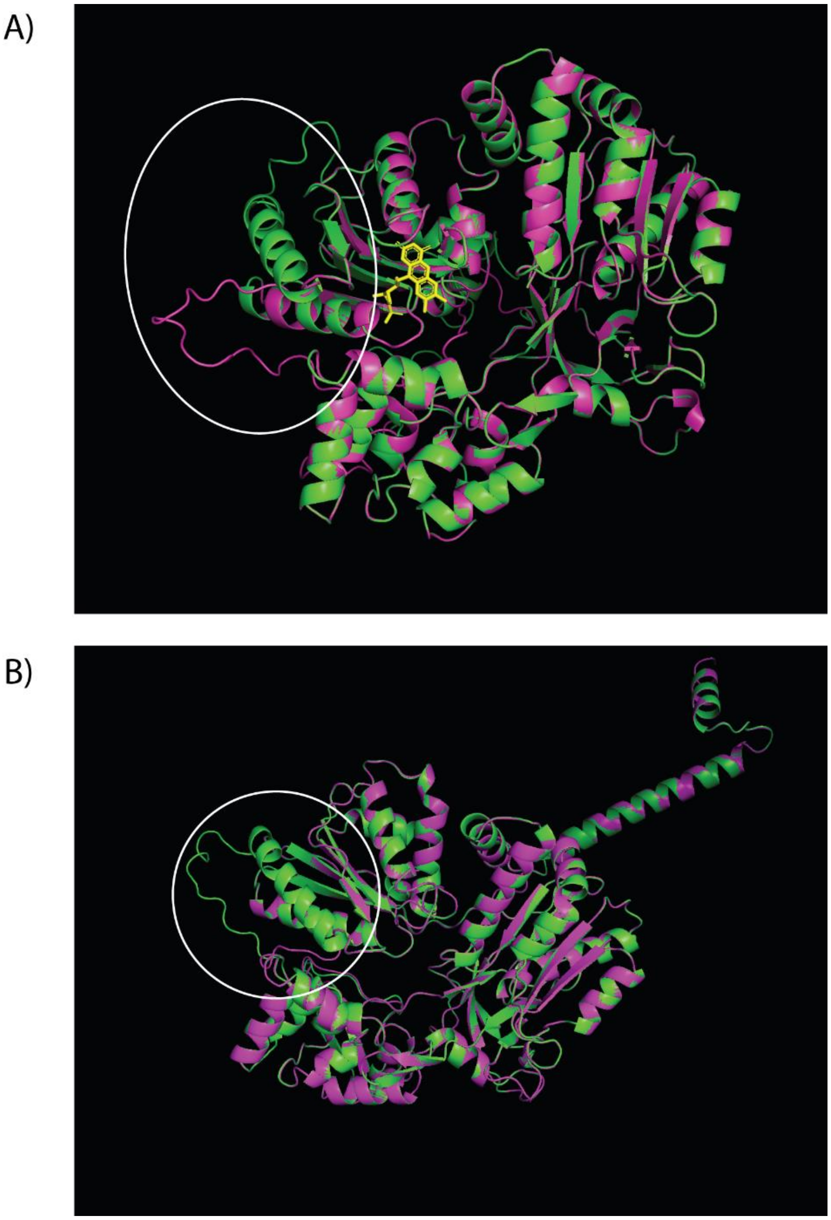

Figure S11. Crystal structure modelling of wild-type LjCPR1 (green) overlapped with non-frameshift mutant LjCPR1 from *Ljcpr1*-KO mutant hairy root line L1-4.2 (magenta) using (A) 5gxu.1.A and (B) A0A0R4J338.1.A as template. The FMN ligand in (A) is shown by yellow color. The white circle indicates the location of the missing α-helix resulted from the missing 24 amino acids on the mutant LjCPR1.

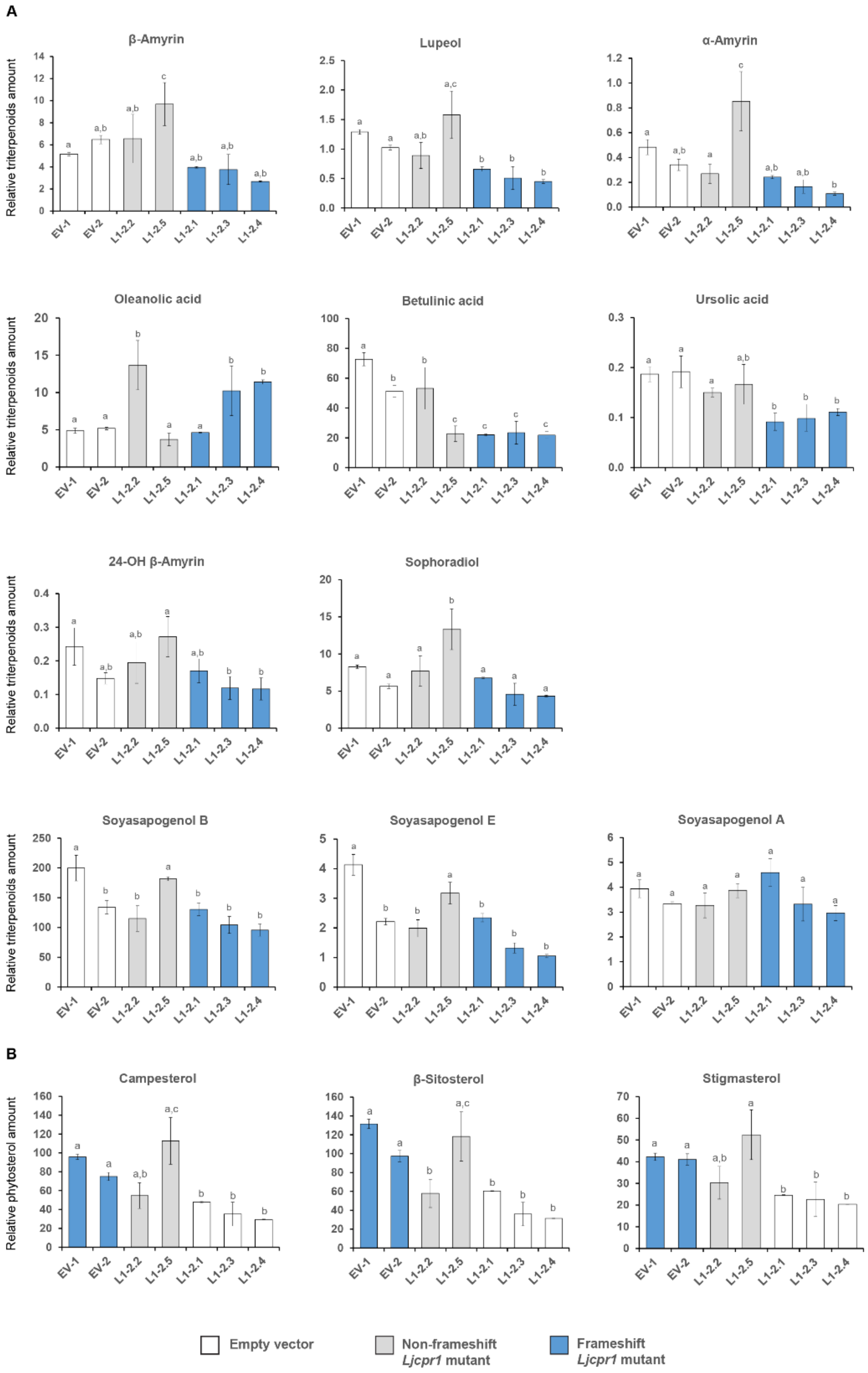

Figure S12. The relative amount of A) triterpenoids and B) phytosterol content hairy root *Ljcpr-1* (target 2B) mutants analyzed by GC-MS.

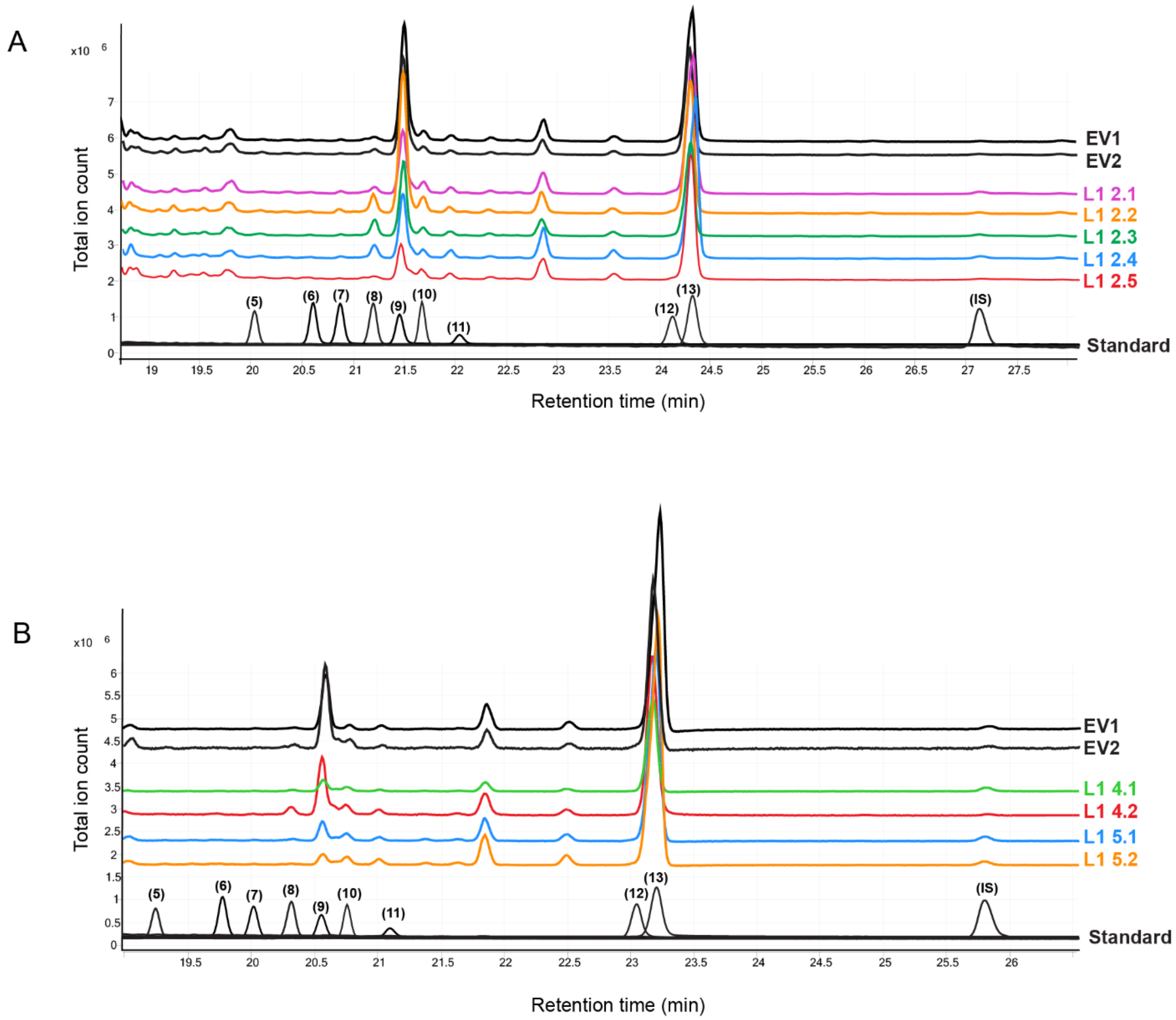

Figure S13. TIC scan of GC-MS chromatogram of *Ljcpr1* knockout hairy root mutants A) target gRNA No. 2, and B) target gRNA No. 4 and 5. Compound numbers are consistent with those in Table S7. The peaks of betulinic acid decreased significantly in *Ljcpr1* frameshift mutants (L1-2.1, 2.3, 2.4, 4.1, 5.1, 5.2).

Table S1. List of accession numbers of CPR genes and amino acid sequences from other plant species used for phylogenetic analysis in this study. Yellow color shows genes not available in NCBI.

Gene	Gene accession number / Gene ID	Protein accession number	Source
Arabidopsis thaliana CPR1 (ATR1)	NM_118585.4	NP_194183.1	NCBI
Arabodipsis thaliana CPR2 (ATR2)	NM_119167.4	NP_194750.1	NCBI
M. truncatula CPR1	XM_003602850.3	XP_003602898.1	NCBI
M. truncatula CPR2	XM_003610061.4	XP_003610109.1	NCBI
G. uralensis CPR1	KY798117.1	AUG98241.1	NCBI
G. uralensis CPR2	MH401048.1	QCZ35624.1	NCBI
Cicer arietinum CPR1	XM_004501597.3	XP_004501654.1	NCBI
Cicer arietinum CPR2	XM_004507801.3	XP_004507858.1	NCBI
Chenopodium quinoa CPR1	XM_021904070.1	XP_021759762.1	NCBI
Chenopodium quinoa CPR2	XM_021867713.1	XP_021723405.1	NCBI
Spinacia oleracea CPR1	XM_021999727.1	XP_021855419.1	NCBI
Spinacia oleracea CPR2	XM_022003966.1	XP_021859658.1	NCBI
Solanum lycopersicum CPR1	XM_004237953.3	XP_004238001.1	NCBI
Solanum lycopersicum CPR2	XM_004242883.4	XP_004242931.1	NCBI
Solanum tuberosum CPR1	XM_006337990.2	XP_006338052.1	NCBI
Solanum tuberosum CPR2	PGSC0003DMT400035801	-	www.plantgdb.org
Artemisia annua CPR1	PKPP01006895.1 (Whole genome sequence)	PWA55016.1	NCBI
Artemisia annua CPR2	EF104642.1	EF104642.1	NCBI
Catharantus roseus CPR2	X69791.1	CAA49446.1	
Oryza sativa CPR2a	CM000134.1	EAZ10065.1	NCBI
Oryza sativa CPR2c	AL606690.3	CAE03554.2	NCBI
Triticum aestivum CPR2a	AJ303373.1	CAC83301.1	NCBI
Triticum aestivum CPR2c	AF123610.1	AAG17471.1	NCBI
Zea Mays CPR2b 1	EU955593.1	ACG27711.1	NCBI
Pseudotsuga menziesii CPR	CAA89837.3	Z49767.3	NCBI
Taxus chinensis CPR	AAX59902.1	AY959320.1	NCBI
Taxus cuspidata CPR	AAT76449.1	AY571340.1	NCBI
Physcomitrella patens CPR	EDQ49310.1	DS545408.1	NCBI
Selaginella moellendorffii CPR	XP_002978784.2	XM_002978738.2	NCBI
Chlamydomonas reinhardtii CPR	XP_042928682.1	XM_043058768.1	NCBI

Table S2. (A) Amino acid and (B) nucleotide sequence identity matrix of LjCPRs from Miyakojima MG-20 and Gifu B-129 ecotype.

(A)

Source	Gene ID/Accession No.	NCBI	Miyakojima (MG20)			Gifu						
		BAG68945.1	Lj1g0009282.1	Lj4g0009595.1	Lj4g0000806.1	LotjaGi1g1v0345200.1	LotjaGi4g1v0301400.3	LotjaGi4g1v0301400.4	LotjaGi4g1v0301400.2	LotjaGi4g1v0301400.1	LotjaGi4g1v0301300.1	LotjaGi4g1v0301300.2
NCBI	BAG68945.1	ID	0.666	**1**	0.916	0.666	**1**	0.349	0.998	0.919	0.919	0.919
Miyakojima (MG20)	Lj1g0009282.1	0.666	ID	0.666	0.638	**1**	0.666	0.282	0.665	0.641	0.641	0.641
	Lj4g0009595.1	**1**	0.666	ID	0.916	0.666	**1**	0.349	0.998	0.919	0.919	0.919
	Lj4g0000806.1	0.916	0.638	0.916	ID	0.638	0.916	0.339	0.917	0.991	0.991	0.991
Gifu	LotjaGi1g1v0345200.1	0.666	**1**	0.666	0.638	ID	0.666	0.282	0.665	0.641	0.641	0.641
	LotjaGi4g1v0301400.3	**1**	0.666	**1**	0.916	0.666	ID	0.349	0.998	0.919	0.919	0.919
	LotjaGi4g1v0301400.4	0.349	0.282	0.349	0.339	0.282	0.349	ID	0.348	0.342	0.342	0.342
	LotjaGi4g1v0301400.2	0.998	0.665	0.998	0.917	0.665	0.998	0.348	ID	0.920	0.920	0.920
	LotjaGi4g1v0301400.1	0.919	0.641	0.919	0.991	0.641	0.919	0.342	0.920	ID	**1**	**1**
	LotjaGi4g1v0301300.1	0.919	0.641	0.919	0.991	0.641	0.919	0.342	0.920	**1**	ID	**1**
	LotjaGi4g1v0301300.2	0.919	0.641	0.919	0.991	0.641	0.919	0.342	0.920	**1**	**1**	ID

(B)

Source	Gene ID	NCBI	Miyakojima (MG20)			Gifu						
		BAG68945.1	Lj1g0009282.1	Lj4g0009595.1	Lj4g0000806.1	LotjaGi1g1v0345200.1	LotjaGi4g1v0301400.3	LotjaGi4g1v0301400.4	LotjaGi4g1v0301400.2	LotjaGi4g1v0301400.1	LotjaGi4g1v0301300.1	LotjaGi4g1v0301300.2
NCBI	**BAG68945.1**	ID	0.668	**1**	0.949	0.668	**1**	0.350	0.999	0.950	0.950	0.950
Miyakojima (MG20)	**Lj1g0009282.1**	0.668	ID	0.668	0.652	**1**	0.668	0.260	0.668	0.653	0.653	0.653
	**Lj4g0009595.1**	**1**	0.668	ID	0.949	0.668	**1**	0.350	0.999	0.950	0.950	0.950
	**Lj4g0000806.1**	0.949	0.652	0.949	ID	0.652	0.949	0.344	0.950	0.995	0.995	0.995
Gifu	**LotjaGi1g1v0345200.1**	0.668	**1**	0.668	0.652	ID	0.668	0.260	0.668	0.653	0.653	0.653
	**LotjaGi4g1v0301400.3**	**1**	0.668	**1**	0.949	0.668	ID	0.350	0.999	0.950	0.950	0.950
	**LotjaGi4g1v0301400.4**	0.350	0.260	0.350	0.344	0.260	0.350	ID	0.349	0.345	0.345	0.345
	**LotjaGi4g1v0301400.2**	0.999	0.668	0.999	0.950	0.668	0.999	0.349	ID	0.951	0.951	0.951
	**LotjaGi4g1v0301400.1**	0.950	0.653	0.950	0.995	0.653	0.950	0.345	0.951	ID	**1**	**1**
	**LotjaGi4g1v0301300.1**	0.950	0.653	0.950	0.995	0.653	0.950	0.345	0.951	**1**	ID	**1**
	**LotjaGi4g1v0301300.2**	0.950	0.653	0.950	0.995	0.653	0.950	0.345	0.951	**1**	**1**	ID

Tables S3. Primer sequences used for qPCR analysis of MeJA-treated *L. japonicus* hairy roots

Gene	Primer Sequence
**Primers for qPCR analysis of *L. japonicus* hairy root**	
LjCPR1_qPCR_Fw	ATGACTTCGAATTCCGATTTGG
LjCPR1_qPCR_Rv	GTCGTCACGATCAGAATCAGC
LjCPR2-1_qPCR_Fw	CGAGAAGCTTAGCGACGAGG
LjCPR2-1_qPCR_Rv	CTCTTCCGCAATCGCCTTG
LjCPR2-2_qPCR_Fw	CGTCGACGAGGCTGAGGTTGAC
LjCPR2-2_qPCR_Rv	GCCACAAGCGCCTTGGCGAATC
LjUBQ_qPCR_Fw	TTCACCTTGTGCTCCGTCTTC
LjUBQ_qPCR_Rv	AACAACAGCACACACAGACAATCC
LjCYP716A51_qPCR_Fw	GTCTTCCCCTCATCACTCCA
LjCYP716A51_qPCR_Rv	TGTCTCTGCGCTATGTCGTC
LjCYP93E1_qPCR_Fw	AGCACTTTGTCAGCGTTCG
LjCYP93E1_qPCR_Rv	ACGGCTTCACCTGTTTTTGA
LjCYP72A61_qPCR_Fw	GTGTGATTGCTACGGTGGTG
LjCYP72A61_qPCR_Rv	GGAGACCCTGCTGCTTCATA
LjbAS_qPCR_Fw	TCACTTACGGTTCTTGGTTCG
LjbAS_qPCR_Rv	CGCCATCACCTCTTTGTGTAG
LjLUS_qPCR_Fw	TATGAGTGGTCAGGGTGCAA
LjLUS_qPCR_Rv	GGGCATGTAAACTAAGCGACA
LjaAS_qPCR_Fw	TGGGCTTTGATGGCTCTAATTC
LjaAS_qPCR_Rv	TTTGCGGCATGATGAAGTGG
LjLAS_qPCR_Fw	GGAACTGAACAAGAACGAGCTCAAG
LjLAS_qPCR_Rv	CCATTTTCCCTCTCAAACTGGAGTC
LjCAS_qPCR_Fw	CTGAAGAGGCTGTGGTAACAACG
LjCAS_qPCR_Rv	CATTGGACCTCCATAATCCCCTG

Table S4. All *LORE1* insertions in the genome of the selected *Ljcpr1* and *Ljcpr2-1* mutant lines (lotus.au.dk)

Mutant name	Plant line ID	Genomic position	Gene ID	Insertion type	Gene annotation
L1-A	30003941	chr1_13956169_R	Lj1g3v1113880	Intronic	PREDICTED: probable inactive receptor kinase At5g58300-like isoform X1
		**chr1_18540324_F**	**Lj1g3v1548790**	**Exonic**	**PREDICTED: NADPH--cytochrome P450 reductase-like isoform X2 gi|502133111|ref|XP_004501654.1|**
		chr0_141891533_F	Lj0g3v0273739	Exonic	PREDICTED: ATP-dependent zinc metalloprotease FTSH 12, chloroplastic-like
L1-B	30059903	**chr1_18538152_R**	**Lj1g3v1548790**	**Exonic**	**PREDICTED: NADPH--cytochrome P450 reductase-like isoform X2 gi|502133111|ref|XP_004501654.1|**
		chr5_23067809_R	Lj5g3v1598550	Exonic	PREDICTED: PH-interacting protein-like gi|502117700|ref|XP_004495905.1|
		chr0_144522704_F	Lj0g3v0278319	Exonic	Lactoylglutathione lyase / glyoxalase I family protein gi|508699005|gb|EOX90901.1|
L2-1A	30037476	**chr4_28883580_R**	**Lj4g3v2107220**	**Exonic**	**cytochrome P450 reductase [*L. japonicus*] gi|197209812|dbj|BAG68945.1|**
		chr2_35390899_F	Lj2g3v2574420	Exonic	Receptor-type tyrosine-protein phosphatase U [Theobroma cacao] gi|508728148|gb|EOY20045.1|
		chr0_125503891_F	-	Intergenic	-
L2-1B	30065390	chr2_11997511_R	Lj2g3v0766630	Intronic	Serine incorporator [*M. truncatula*] gi|357500415|ref|XP_003620496.1|
		chr0_20652508_R	Lj0g3v0061569	Intronic	PREDICTED: ras-related protein RABH1b-like [Cicer arietinum] gi|502152574|ref|XP_004508989.1|
		**chr4_28884617_F**	**Lj4g3v2107220**	**Exonic**	**cytochrome P450 reductase [*L. japonicus*] gi|197209812|dbj|BAG68945.1|**
		chr0_166260633_F	Lj0g3v0318339	Exonic	-

Table S5. *LORE1* genotyping primer sequences

Gene	Primer Sequence	Additional description
**Primers for LORE1 mutant PCR genotyping**		
3941_LjCPR1_Fw	TCCACCATGTATCCAAACACCCCACA	L1-A F
3941_LjCPR1_Rv	GAGGCGAAGACAGCAAATCGACGC	L1-A R
59903_LjCPR1_Fw	TTTTTGGCAATCCCTCGTTCCGGT	L1-B F
59903_LjCPR1_Rv	GCCTGTTACCCAGGGCAAAAACTCC A	L1-B R
37476_LjCPR2-1_Fw	AGGCGATTGCGGAAGAGGCAAAAG	L2-1A F
37476_LjCPR2-1_Rv	TCCACTTCAATGGCGACCTGTGTCA	L2-1A R
65390_LjCPR2-1_Fw	GACGTTGGAAGGGTCGAGTGTGCC	L2-1B F
65390_LjCPR2-1_Rv	CAGCTGCGCTCGTTTTCGATTGGT	L2-1B R
P2 *LORE1*	CCATGGCGGTTCCGTGAATCTTAGG	*LORE1* insertion specific reverse primer
chr0_141891533_F	GCTGCGAAAATGCATGCCAGTCAA	Other insertion in L1-A line
chr0_141891533_R	GGGCACTTCAAAACCTGTAGCTGCC CT	Other insertion in L1-A line
chr0_144522704_F	GGAAGATTTTCCAGCGAGGGACGA	Other insertion in L1-B line
chr0_144522704_R	GGTGGACCAGGTGTTCTTCCACGA	Other insertion in L1-B line
chr5_23067809_F	CAATAGACAGCCACACGGTGACCCC	Other insertion in L1-B line
chr5_23067809_R	TCCACCTAATCAAGACTGGTACTGA GGCA	Other insertion in L1-B line

Table S6. Primer sequences used to construct gRNAs targeting *LjCPR1* gene

Gene	Primer Sequence	Additional description
**Primers for gRNA construct targeting *LjCPR1* gene**		
F2_tgRNA_2A_LjCPR1	ttgggtctcgTGCAGCTGGCTTCCAATCACCC ATTGTTTTAGAGCTAGAAATAGCA	
R2_tgRNA_2B_LjCPR1	ttgggtctccAAACTCGTTTACAGCCTCGTTA TTCTGCACCAGCCGGGAATCGAA	
F2_tgRNA_LjCPR1-4A	ttgggtctcgTGCAGAGGATGGCACTCCCCT AGGGTTTTAGAGCTAGAAATAGCA	
R2_tgRNA_LjCPR1-4B	ttgggtctccAAACTGCACACTGCGCACTGC ATTCTGCACCAGCCGGGAATCGAA	
F2_tgRNA_LjCPR1-5A	ttgggtctcgTGCAGTCACTTCAAGTAGACTT CTCGTTTTAGAGCTAGAAATAGCA	
R2_tgRNA_LjCPR1-5B	ttgggtctccAAACCGTTATTATTCTATTTCA TCTGCACCAGCCGGGAATCGAA	
**Primers for checking CRISPR-Cas9 mutation in *LjCPR1***		
LjCPR1_F_982	GGAGACCATGTGGGTGTTTATGCTG	To check target gRNA LjCPR1-4
LjCPR1_R_1222	GAGCAGCTAATGCAACTAGAGCAGC	To check target gRNA LjCPR1-4
LjCPR1_F_1208	GCTGCTCTAGTTGCATTAGCTGC	To check target gRNA LjCPR1-2
LjCPR1_R_1485	CGTTGGACCACAAACCAAGGCACAAG	To check target gRNA LjCPR1-2 or LjCPR1-5
LjCPR1_F_1198	GCTGCTCTAGTTGCATTAGCTGCTC	To check target gRNA LjCPR1-5

Note: The nucleotide sequence of *LjCPR1* gene used in this study has been submitted to the DNA Data Bank of Japan (DDBJ) under the accession numbers of LC744374.

Table S7. List of m/z values for the target ion and qualifier ion used in GC-MS analysis

**Compounds**	**Target ion**	**Qualifier ion**
β-amyrin **(1)**	218	203
α-amyrin **(2)**	218	203
Lupeol **(3)**	189	203
24-OH β-amyrin **(4)**	218	203
Oleanolic acid **(8)**	203	320
Betulinic acid **(9)**	189	203
Sophoradiol **(10)**	306	291
Ursolic acid **(11)**	203	320
Soyasapogenol E **(12)**	232	278
Soyasapogenol B **(13)**	306	291
Soyasapogenol A **(14)**	394	278
Campesterol **(15)**	382	400
β-sitosterol **(16)**	397	400
Stigmasterol **(17)**	395	400
Asiatic Acid (IS) **(18)**	320	203

Table S8. Co-expression analysis of closely correlated genes with CPR class I and II in *L. japonicus*

No.	***LjCPR1*** (Ljwgs_006504.2_at)			***LjCPR2*** (Ljwgs_068084.1_at)		
	**Probeset**	**PCC value**	**Initial annotation during chip design**	**Probeset**	**PCC value**	**Initial annotation during chip design**
1	TM1224.12_at	0.75	*L. japonicus* similar to At3g08580: adenylate translocator	Ljwgs_109412.1_at	0.68	*L. japonicus* similar to At3g51480: glutamate receptor like protein → defense against pathogens, reproduction, control of stomata aperture and light signal transduction
2	TM1224.12.1_at	0.75	*L. japonicus* similar to At3g08580: adenylate translocator	Ljwgs_016866.2_at	0.67	*L. japonicus* similar to At5g58870: cell division protein - like
3	chr1.TM0430.17.1_at	0.75	*L. japonicus* similar to At5g48900: pectate lyase	TC10072_at	0.66	homologue to UP|Q863B4 (Q863B4) Trefoil factor 3, partial (15%)
4	chr2.CM0056.38_at	0.72	*L. japonicus* similar to At1g60070: hypothetical protein	chr1.CM0591.55_at	0.66	*L. japonicus* similar to Q40983: (Q40983) METALLOENDOPEPTIDASE: amyloid precursor protein catabolic process
5	chr1.CM0105.95_at	0.72	*L. japonicus* similar to At3g54770: RNA binding protein - like	Ljwgs_051871.1_at	0.66	*L. japonicus* similar to At1g50360: myosin, putative
6	TM0759.8_at	0.71	*L. japonicus* similar to At2g26640: putative beta-ketoacyl-CoA synthase	Ljwgs_023382.1_at	0.65	*L. japonicus* similar to O04434: (O04434) PUTATIVE NADPH-CYTOCHROME P450 REDUCTASE

7	Ljwgs_081701.1_at	0.71	*L. japonicus* similar to At4g12420: pollen-specific protein - like predicted GPI-anchored protein	Ljwgs_043693.1_at	0.65	*L. japonicus* similar to At2g32400: ionotropic glutamate receptor (GLR5)
8	Ljwgs_022220.1_at	0.71	*L. japonicus* similar to At4g00710: unknown protein	Ljwgs_074438.1_at	0.65	*L. japonicus* similar to At1g30360: ERD4 protein (ERD4: Early-responsive to dehydration stress protein)
9	chr5.CM0328.80_at	0.71	*L. japonicus* similar to At5g08680: H+-transporting ATP synthase beta chain (mitochondrial) - like protein	chr1.CM0591.54_at	0.64	*L. japonicus* similar to At5g42390: pitrilysin
10	Ljwgs_089550.1_at	0.71	*L. japonicus* similar to At4g28650: receptor protein kinase-like protein	Ljwgs_058241.1_at	0.63	*L. japonicus* similar to At2g39190: ABC transporter like protein

Supplementary File 1

Amino acid sequences of mutant LjCPR1 in *Ljcpr1-KO* mutant in hairy roots

- **Wild-type LjCPR1**

Wild-type LjCPR1 (691aa)

MTSNSDLVRTIESVLGVSLGDSVSDSLILIVTTSVAIIIGLLVVLWKKSSDRSKEVKPLVVPKLLVN
DEEEDDVASGKTKVTVFFGTQTGTAEGFAKALAEEIKARYEKAAVKVVDLDDYAMDDDQYEEK
LKKETLAFFMLATYGDGEPTDNAARFYKWFTEGKGERSETWLKQLTYGVFALGNRQYEHFNK
VGKVVDDDLSEQGAKRLVPVGLGDDDQSIEDDFSAWKESLWPELDQLLQDEDDLKTVSTPYR
AAIPEYRVVIHDPAATLPYDSHLNMANGNAVFDIHHPCRGNVAVRKELHKPESDRSCIHLEFDIS
GIGITYETGDHVGVYAENCAETVEEAGELLGQNLELLFSLHTDNEDGTPLGGSLPPTFPGPCTL
RTALSRYTDLLTPPRKAALVALAAHVSEPSEAEKLKFLLSPQGKDEYSKWVIGSQRSLLEVMAE
FPSAKPPLGVFFAAIAPRLQPRYYSISSSPRYAPQRVHVTCALVCGPTPTGRIHKGVCSTWMKD
AIPLEENRDCSWAPIFIRPSNFKLPADHSIPIIMVGPGTGLAPFRGFLQERFALKEDGIELGPAILF
FGCRNRRMDFIYEDELNNFLEQGSLSELIVAFSREGSEKEYVQHKMMDKAAYLWSLISQGAYL
YVCGDAKGMARDVHHTLHTIVQQQENVESSKAEAIVKKLQLDGRYLRDVW*

- **L1-4.1**

-37bp (1107st – 1143rd) → frameshift and early termination at 381st amino acid (381 aa)

MTSNSDLVRTIESVLGVSLGDSVSDSLILIVTTSVAIIIGLLVVLWKKSSDRSKEVKPLVVPKLLVN
DEEEDDVASGKTKVTVFFGTQTGTAEGFAKALAEEIKARYEKAAVKVVDLDDYAMDDDQYEEK
LKKETLAFFMLATYGDGEPTDNAARFYKWFTEGKGERSETWLKQLTYGVFALGNRQYEHFNKI
GKVVDDDLSEQGAKRLVPVGLGDDDQSIEDDFSAWKESLWPELDQLLQDEDDLKTVSTPYRA
AIPEYRVVIHDPAATLPYDSHLNMANGNAVFDIHHPCRGNVAVRKELHKPESDRSCIHLEFDISG
IGITYETGDHVGVYAENCAETVEEAGELLGQNLELLFSLHTDNEDGTPLHCALHYRVIQIS*

-2bp (1105th – 1107th) → frameshift and early termination at 409th amino acid (409 aa)

MTSNSDLVRTIESVLGVSLGDSVSDSLILIVTTSVAIIIGLLVVLWKKSSDRSKEVKPLVVPKLLVN
DEEEDDVASGKTKVTVFFGTQTGTAEGFAKALAEEIKARYEKAAVKVVDLDDYAMDDDQYEEK
LKKETLAFFMLATYGDGEPTDNAARFYKWFTEGKGERSETWLKQLTYGVFALGNRQYEHFNKI
GKVVDDDLSEQGAKRLVPVGLGDDDQSIEDDFSAWKESLWPELDQLLQDEDDLKTVSTPYRA
AIPEYRVVIHDPAATLPYDSHLNMANGNAVFDIHHPCRGNVAVRKELHKPESDRSCIHLEFDISG
IGITYETGDHVGVYAENCAETVEEAGELLGQNLELLFSLHTDNEDGTPRRFSATYIPRSLHTAHC
IIALYRSLDPPAKGCSSCISCSCF*

- **L1-4.2**

-72bp (222nd - 246th) → non-frameshift, only loss 24 amino acids (666 aa)

MTSNSDLVRTIESVLGVSLGDSVSDSLILIVTTSVAIIIGLLVVLWKKSSDRSKEVKPLVVPKLLVN
DEEEDDVASGKTKVTVFFGTQTGTAEGFAKALAEEIKARYEKAAVKVVDLDDYAMDDDQYEEK
LKKETLAFFMLATYGDGEPTDNAARFYKWFTEGKGERSETWLKQLTYGVFALGNRQYEHFNKI
GKVVDDDLSEQGAKRLVPVGLGDDDQSIKTVSTPYRAAIPEYRVVIHDPAATLPYDSHLNMAN
GNAVFDIHHPCRGNVAVRKELHKPESDRSCIHLEFDISGIGITYETGDHVGVYAENCAETVEEA
GELLGQNLELLFSLHTDNEDGTPLGGSLPPTFPGPCTLRTALSRYTDLLTPPRKAALVALAAHV

SEPSEAEKLKFLLSPQGKDEYSKWVIGSQRSLLEVMAEFPSAKPPLGVFFAAIAPRLQPRYYSIS
SSPRYAPQRVHVTCALVCGPTPTGRIHKGVCSTWMKNAIPLEENRDCSWAPIFIRPSNFKLPAD
HSIPIIMVGPGTGLAPFRGFLQERFALKEDGIELGPAILFFGCRNRRMDFIYEDELNNFLEQGSLS
ELIVAFSREGSEKEYVQHKMMDKAAYLWSLISQGAYLYVCGDAKGMARDVHHTLHTIVQQQEN
VESSKAEAIVKKLQLDGRYLRDVW

-37bp (1107st – 1143rd) → frameshift and early termination at 381st amino acid (381 aa)

MTSNSDLVRTIESVLGVSLGDSVSDSLILIVTTSVAIIIGLLVVLWKKSSDRSKEVKPLVVPKLLVN
DEEEDDVASGKTKVTVFFGTQTGTAEGFAKALAEEIKARYEKAAVKVVDLDDYAMDDDQYEEK
LKKETLAFFMLATYGDGEPTDNAARFYKWFTEGKGERSETWLKQLTYGVFALGNRQYEHFNKI
GKVVDDDLSEQGAKRLVPVGLGDDDQSIEDDFSAWKESLWPELDQLLQDEDDLKTVSTPYRA
AIPEYRVVIHDPAATLPYDSHLNMANGNAVFDIHHPCRGNVAVRKELHKPESDRSCIHLEFDISG
IGITYETGDHVGVYAENCAETVEEAGELLGQNLELLFSLHTDNEDGTPLHCALHYRVIQIS*

- **L1-5.1**

-22bp (1406th – 1427nd) → frameshift and early termination at 485th amino acid (485 aa)

MTSNSDLVRTIESVLGVSLGDSVSDSLILIVTTSVAIIIGLLVVLWKKSSDRSKEVKPLVVPKLLVN
DEEEDDVASGKTKVTVFFGTQTGTAEGFAKALAEEIKARYEKAAVKVVDLDDYAMDDDQYEEK
LKKETLAFFMLATYGDGEPTDNAARFYKWFTEGKGERSETWLKQLTYGVFALGNRQYEHFNKI
GKVVDDDLSEQGAKRLVPVGLGDDDQSIEDDFSAWKESLWPELDQLLQDEDDLKTVSTPYRA
AIPEYRVVIHDPAATLPYDSHLNMANGNAVFDIHHPCRGNVAVRKELHKPESDRSCIHLEFDISG
IGITYETGDHVGVYAENCAETVEEAGELLGQNLELLFSLHTDNEDGTPLGGSLPPTFPGPCTLR
TALSRYTDLLTPPRKAALVALAAHVSEPSEAEKLKFLLSPQGKDEYSKWVIGSQRSLLEVMAEF
PSAKPPLGVFFAAIAPRLQPRYYSISSFLGMPHKGYM*

-1bp (1408th) → frameshift and early termination at 485th amino acid (485 aa)

MTSNSDLVRTIESVLGVSLGDSVSDSLILIVTTSVAIIIGLLVVLWKKSSDRSKEVKPLVVPKLLVN
DEEEDDVASGKTKVTVFFGTQTGTAEGFAKALAEEIKARYEKAAVKVVDLDDYAMDDDQYEEK
LKKETLAFFMLATYGDGEPTDNAARFYKWFTEGKGERSETWLKQLTYGVFALGNRQYEHFNKI
GKVVDDDLSEQGAKRLVPVGLGDDDQSIEDDFSAWKESLWPELDQLLQDEDDLKTVSTPYRA
AIPEYRVVIHDPAATLPYDSHLNMANGNAVFDIHHPCRGNVAVRKELHKPESDRSCIHLEFDISG
IGITYETGDHVGVYAENCAETVEEAGELLGQNLELLFSLHTDNEDGTPLGGSLPPTFPGPCTLR
TALSRYTDLLTPPRKAALVALAAHVSEPSEAEKLKFLLSPQGKDEYSKWVIGSQRSLLEVMAEF
PSAKPPLGVFFAAIAPRLQPRIILFHPLLGMP*

- **L1-5.2**

-22bp (1389th – 1422nd) → frameshift and early termination at 474th amino acid (474 aa)

MTSNSDLVRTIESVLGVSLGDSVSDSLILIVTTSVAIIIGLLVVLWKKSSDRSKEVKPLVVPKLLVN
DEEEDDVASGKTKVTVFFGTQTGTAEGFAKALAEEIKARYEKAAVKVVDLDDYAMDDDQYEEK
LKKETLAFFMLATYGDGEPTDNAARFYKWFTEGKGERSETWLKQLTYGVFALGNRQYEHFNKI
GKVVDDDLSEQGAKRLVPVGLGDDDQSIEDDFSAWKESLWPELDQLLQDEDDLKTVSTPYRA
AIPEYRVVIHDPAATLPYDSHLNMANGNAVFDIHHPCRGNVAVRKELHKPESDRSCIHLEFDISG
IGITYETGDHVGVYAENCAETVEEAGELLGQNLELLFSLHTDNEDGTPLGGSLPPTFPGPCTLR

TALSRYTDLLTPPRKAALVALAAHVSEPSEAEKLKFLLSPQGKDEYSKWVIGSQRSLLEVMAEF
PSAKPPLGVFFAAIAPLLGMPHKGYM*

-1bp (1408th) → frameshift and early termination at 485th amino acid (485 aa)

MTSNSDLVRTIESVLGVSLGDSVSDSLILIVTTSVAIIIGLLVVLWKKSSDRSKEVKPLVVPKLLVN
DEEEDDVASGKTKVTVFFGTQTGTAEGFAKALAEEIKARYEKAAVKVVDLDDYAMDDDQYEEK
LKKETLAFFMLATYGDGEPTDNAARFYKWFTEGKGERSETWLKQLTYGVFALGNRQYEHFNKI
GKVVDDDLSEQGAKRLVPVGLGDDDQSIEDDFSAWKESLWPELDQLLQDEDDLKTVSTPYRA
AIPEYRVVIHDPAATLPYDSHLNMANGNAVFDIHHPCRGNVAVRKELHKPESDRSCIHLEFDISG
IGITYETGDHVGVYAENCAETVEEAGELLGQNLELLFSLHTDNEDGTPLGGSLPPTFPGPCTLR
TALSRYTDLLTPPRKAALVALAAHVSEPSEAEKLKFLLSPQGKDEYSKWVIGSQRSLLEVMAEF
PSAKPPLGVFFAAIAPRLQPRIILFHPLLGMP*
